# Body Fluid Cytokine Levels in Mild Cognitive Impairment and Alzheimer’s Disease: a Comparative Overview

**DOI:** 10.1007/s12035-014-8657-1

**Published:** 2014-02-25

**Authors:** Frederic Brosseron, Marius Krauthausen, Markus Kummer, Michael T. Heneka

**Affiliations:** 1 German Center for Neurodegenerative Diseases (DZNE), Bonn, Germany; 2 Clinic and Polyclinic for Neurology, Clinical Neuroscience Unit, University Hospital Bonn, Bonn, Germany; 3 German Center for Neurodegenerative Diseases (DZNE), Clinical Neuroscience Unit, Clinic and Polyclinic for Neurology, Sigmund-Freud-Str. 25, 53127 Bonn, Germany

**Keywords:** Neuroinflammation, Cytokines, Serum, Cerebrospinal fluid, Mild cognitive impairment, Alzheimer’s disease

## Abstract

This article gives a comprehensive overview of cytokine and other inflammation associated protein levels in plasma, serum and cerebrospinal fluid (CSF) of patients with Alzheimer’s disease (AD) and mild cognitive impairment (MCI). We reviewed 118 research articles published between 1989 and 2013 to compare the reported levels of 66 cytokines and other proteins related to regulation and signaling in inflammation in the blood or CSF obtained from MCI and AD patients. Several cytokines are evidently regulated in (neuro-) inflammatory processes associated with neurodegenerative disorders. Others do not display changes in the blood or CSF during disease progression. However, many reports on cytokine levels in MCI or AD are controversial or inconclusive, particularly those which provide data on frequently investigated cytokines like tumor necrosis factor alpha (TNF-α) or interleukin-6 (IL-6). The levels of several cytokines are possible indicators of neuroinflammation in AD. Some of them might increase steadily during disease progression or temporarily at the time of MCI to AD conversion. Furthermore, elevated body fluid cytokine levels may correlate with an increased risk of conversion from MCI to AD. Yet, research results are conflicting. To overcome interindividual variances and to obtain a more definite description of cytokine regulation and function in neurodegeneration, a high degree of methodical standardization and patients collective characterization, together with longitudinal sampling over years is essential.

**Electronic supplementary material:**

The online version of this article (doi:10.1007/s12035-014-8657-1) contains supplementary material, which is available to authorized users.

## Introduction

Cytokines are small signaling proteins with a large spectrum of functions in inflammatory processes and immune system regulation [1]. Therefore, they have been investigated in the context of neuroinflammation, a process accompanying and probably contributing to pathology in several neurodegenerative diseases including Alzheimer’s disease (AD) or Parkinson’s disease (PD) [2–5]. One key feature of neuroinflammation is activation of microglia, which includes local changes of cytokine expression [2, 3]. Additionally, systemic levels of cytokines may rise in response to aging and stress, known risk factors for neurodegeneration [6–8]. Susceptibility for inflammation rises with age and might be enhanced by each inflammatory event [9]. Furthermore, chronic inflammation and the delirium accompanying severe systemic infection have been shown to be risk factors for AD in the elderly, and vice versa, several risk factors for AD are also inducers of systemic inflammation [10–13]. As a consequence, levels of cytokines, their receptors and other proteins associated with immune responses in blood and CSF of AD patients have been frequently investigated to uncover mechanisms of neuroinflammation in dementia or in the context of biomarker research. However, much of the data obtained from different studies is controversial. Here, we give a comprehensive overview of published research in this field and discuss possible reasons behind the conflicting observations.

## Results

### Literature Overview

We included 118 PubMed-listed articles providing data explicitly on levels of immune signaling proteins—primarily cytokines and their receptors—in serum, plasma or CSF of patients with diagnosed MCI or AD in comparison to unaffected control groups. We excluded studies on cytokine levels in human or murine brain tissue, cytokine production by lymphocytes, cytokine polymorphisms or cytokine levels in other neurodegenerative diseases, like PD or frontotemporal dementia. In total, the 118 articles reported data on 66 cytokines, cytokine receptors and other proteins induced by cytokines or otherwise associated with inflammatory signaling and regulation.

Table 1 gives a short summary of literature features: In general, about one third of the articles investigated MCI or other dementia types additional to AD. Plasma, serum and CSF were used in equal terms, and the most frequent method for cytokine determination was singleplex enzyme-linked immunosorbent assay (ELISA). By the last decade, multiplex assays and cytokine arrays were used with increasing frequency. A variety of cognition testing methods and diagnostic criteria were used in the different studies, although most articles noted the use of the National Institute of Neurological and Communicative Disorders and Stroke and the Alzheimer’s Disease and Related Disorders Association (NINCDS-ADRDA) criteria and mini-mental state examination (MMSE) for patient characterization [14, 15]. Supplementary 1 contains a more detailed description of the reviewed articles contents, investigated proteins and used methods.

**Table 1:** Characteristics of reviewed articles on cytokine levels in AD and MCI. The table lists investigated disease type, diagnostic criteria/tests, sample types and methods of the reviewed 118 articles (Supplementary 1). Note that some articles investigated more than one disease or body fluid or used more than a single method. Roughly 1/3 of articles investigated MCI or other dementia types additionally to AD. Most studies reported use of at least one class of diagnostic criteria and one type of cognitive testing. Plasma, serum and CSF were used in equal terms, and the most frequent method was singleplex ELISA, followed especially in the last decade by multiplex assays and cytokine arrays. The percentages reflect the respective proportion assessing the respective features

Disease type	96 %	Alzheimer’s disease
	38 %	Mild cognitive impairment
	27 %	Other dementia or neurological disease
Diagnostic criteria / tests	76 %	NINCDS-ADRDA
	73 %	MMSE
	37 %	DSM-IV
	16 %	DSM-IIIR
	12 %	CDR
	33 %	Other
Sample type	38 %	Plasma
	40 %	Serum
	37 %	CSF
Methods	77 %	ELISA (singleplex)
	8 %	Multiplex assay
	4 %	Cytokine array
	3 %	Western blot
	4 %	Cell-based bioassays
	6 %	Immunodiffusion (solemnly for quantification of ACT)
	4 %	Other methods (radioimmunoassay, immunoephelometry, qRT-PCR)

### General Observations

A brief overview of the described regulations of different cytokines and inflammation associated proteins is given in Table 2. A list of observed effects and used methods for each protein is given in Supplementary 2. Above all, there is a tendency that with growing number of research papers on a particular cytokine there is also an increase in contradictions. For instance, the most frequently investigated cytokines, tumor necrosis factor alpha (TNF-α) and interleukin-6 (IL-6), described in 20–25 % of articles, are reported as upregulated, not regulated or downregulated in the blood or CSF of AD patients (see below, Table 2 and Supplementary 2).

**Table 2:** Regulation of cytokines and inflammation associated proteins in serum/plasma and CSF of AD and MCI patients

Described regulation	Serum/plasma		CSF	
	MCI	AD	MCI	AD
↑ Upregulation	BDNF, IL-1β, MIF, MIP-4, RANTES	CTACK, FGF1, MIF, MIG, sCD40, SCF, VEGF	IL-8, IL-10, MIF, MIG, MIP-4α^a^, sTNF-RII	FGF1, IL-11, IL-18
↑ Upregulation + → No regulation	ICAM-1, IFN-α, TNF-α	ACT, ANG-2, IFN-α, IFN-γ, IL-1β, IL-10, IL-11, IL-18^a^, MIP-1α, sTNF-RI, VCAM-1	MCP-1^a^	ACT, IL-1β, IL-1RII, IL-8, IP-10, MCP-1^a^, VEGF
→ No regulation	ACT, ANG-2, β-NGF, CD40L, CTACK, EGF, G-CSF, Eotaxin, GDNF, GRO-α, HGF, IL-1α, IL-1RII, IL-2R, IL-3, IL-6, IL-10, IL-11, IL-12, IL-16, IL-18, IP-10, LIF, M-CSF, MCP-1, MCP-3, MCP-4, MIG, MIP-1δ, MIP-4α, PDGF-BB, sCD40^a^, SCF, SCGF, SDF-1α, sTNF-RI^a^, sTNF-RII, TRAIL, VCAM-1	β-NGF, E-Selectin, GM-CSF, GRO-α, HGF, IGFBO-6, IL-1RA, IL-1RII, IL-2, IL-2R, IL-7, IL-12^a^, IL-16^a^, IP-10, LIF, MIP-4, sTNF-RII, TRAIL, TRAIL-R4	BDNF, Eotaxin, IL-1β, IL-1RII, MCP-4	β-NGF, FGF2, GDNF, GM-CSF, HGF, IFN-γ, IL-1RA, IL-2, IL-2R, IL-10, IL-12^a^, M-CSF, MIP-1α, SDF-1α, sTNF-RI, sTNF-RII
→ No regulation + ↓ Downregulation	IL-8	G-CSF, IL-1α, IL-6R, MCP-3, SDF-1α		BDNF, IL-6R
↓ Downregulation	IGF-1	G-CSF, IL-1α, IL-6R, MCP-3, P-selectin, SDF-1α	IL-7, M-CSF, TNF-α, TGF-β, VEGF	
↑ Upregulation + → No regulation + ↓ Downregulation		BDNF^a^, CRP, EGF, GDNF, ICAM-1, IL-3, IL-6^a^, IL-8, M-CSF, MCP-1, PDGF-BB, RANTES, TNF-α, TGF-β^a^		IL-6, TNF-α, TGF-β

Overview of the results of the reviewed articles, separated by observed protein expression regulations for serum/plasma and CSF as well as MCI and AD. For several investigated proteins, multiple directions of regulation are described in different articles. For details on synonyms, frequency of effect observation and used methods, see Supplementary 2

^a^Proteins for which disease progression-dependent regulation is described

One explanation for the conflicting results could be differences between the technical approaches of the studies. However, methodological differences alone may not be the solemn source of the variances, as many of the studies used comparable methods: Over 75 % of reviewed articles obtained results from singleplex ELISA using recombinant protein standards for absolute quantification of cytokines (Table 1). It is possible that different ELISA kits do not give identical absolute values of the same analyte [16]. Yet, this cannot explain why different studies reported cytokine levels in AD patients to be higher, unchanged or lower when comparing to control groups. Further, studies using cytokine array technology in a methodological comparable manner also did not provide reproducible results, which indicates that not only technical differences cause conflicting results in cytokine analysis [17–19]. The same applies for other multiplex platforms (e.g., Luminex® platform): Despite high methodical similarities, there were considerable differences in sensitivity, specificity and composition of biofluids-based multianalyte patterns for differentiation between MCI and AD patients and controls [20–25].

Taken together, these observations point to other critical factors, like patient collective composition and patient characterization. For example, it has been shown that cytokine profiles correlate to amyloid burden or APOE genotype, which might be of particular importance for the investigation of such proteins in AD [21, 26]. In this context, it is interesting that in some articles AD patient collectives were subdivided by severity of disease. These reports found differences in cytokine levels between mild, modest or severe AD, e.g., studies by Motta et al., Baranowska-Bik et al., Galimberti et al. [27–29]. Other studies outlined correlations between cytokine levels and disease risk, progression or MCI to AD conversion [27, 29–48]. Yet, a recent meta-analysis of Koyama et al. came to the conclusion that elevation of peripheral cytokine levels is a modest risk factor for neurodegeneration in general, but unspecific for AD [49].

In many studies, strongest upregulation of cytokines was observed in patients with mild AD indicating that cytokine signaling might primarily play a role in the intermediate stages of the disease. On the contrary, patients with advanced AD showed less strong upregulation of cytokines or no differences compared to controls. This might explain why in AD patient collectives, which did not discriminate for disease progression state, no differences to controls or simply higher variances in the AD cohort were observed. Unfortunately, only few studies provide data on disease duration, disease severity or results of neuropsychological examinations like MMSE, which makes it difficult to compare these studies.

Another interesting observation is that some cytokines, especially those apparently not regulated in AD (e.g., interleukin-2, IL-2) where less controversial between studies than cytokines frequently reported to be regulated in any direction (like TNF-α, see Supplementary 2). Thus, the latter still provide interesting research targets, especially under the consideration that subgrouping of patients might provide improved insights into cytokine regulation in AD. In the following, we will give a more detailed description of the regulation of selected cytokines:

### TNF-α

TNF-α is one of the most frequently investigated cytokines. From the 118 articles included, 13 articles describe upregulation, 5 downregulation, and 15 no regulation of TNF-α levels in plasma or serum of AD patients in comparison to control groups [2, 17–19, 28, 32, 33, 35, 50–73]. In an attempt to reduce these variances, we focused on ten articles which report absolute values of plasma or serum TNF-α concentration as obtained by ELISA, include patient group sizes of *n* > 10 and use the MMSE as an estimate of disease severity [18, 28, 33, 35, 50, 52, 64, 68, 69, 74]. Among these ten studies, six report no regulation and four modest upregulation of TNF-α in blood, the latter mostly in patients with severe AD. This might point to disease-state-dependent changes of TNF-α blood levels. Furthermore, the mean values for TNF-α in blood of controls range from 0.7 to 23.0 pg/ml between the studies, pointing towards interassay variances. Also, all studies show high interindividual variances and overlaps between patient and control groups.

Studies analyzing TNF-α in the CSF of AD patients are smaller in number but reflect the same picture: three studies report upregulation of TNF-α, one downregulation and five no regulation [2, 3, 58, 65, 75–79]. In MCI patients, two studies report upregulation and three no regulation in plasma or serum, whereas one study reports downregulation in CSF [3, 19, 32, 51, 52, 63]. The variances between the studies are therefore not limited to blood values.

As studies which reported increased TNF-α-levels often investigated patients with severe AD, it is possible that the levels of this cytokine increase slightly but continuously over the time course of the disease. It is also possible that TNF-α is only upregulated in subgroups of patients which have yet to be defined, e.g., patients suffering from neuroinflammation in addition to a neurodegenerative process.

### TNF Receptors

A different picture is drawn for soluble variants of the TNF receptors (sTNF-RI and sTNF-RII). The levels of both receptors are mostly reported as unchanged in the blood or CSF of AD patients in comparison to controls [32, 33, 50, 65, 75, 80, 81]. For MCI patients, however, data are controversial [32, 80, 82]. Follow up-studies show correlations of TNF receptor levels with risk of MCI to AD conversion [32, 83]. It is possible that individuals with TNF receptor expression in the upper tertile are at increased risk of developing AD. Yet, the observed differences are too small to be used as reliable biomarkers.

### Soluble CD40 and CD40 Ligand

Another member of the TNF receptor superfamily, soluble CD40 (sCD40), is reported to be regulated in AD in a remarkably congruent manner: Three studies describe the elevation of sCD40 plasma levels in AD patients [84–86]. A fourth article by Buchhave et al. reports that levels of sCD40 positively correlate to risk of MCI to AD conversion [38]. Despite variances in effect strength between the studies, sCD40 might be an interesting target for biomarker research, especially since it has not been investigated in CSF of AD or MCI patients. Plasma levels of its binding partner CD40 ligand (CD40L) are described as not regulated in MCI patients and as upregulated in AD patients and might therefore represent another biomarker candidate [38, 84].

### IL-1β

IL-1β is another frequently investigated target in AD, whereas only few reports describe levels in MCI. Interestingly, IL-1β is mostly described as not regulated in CSF of AD patients, while approximately 50 % of reports on serum or plasma levels describe upregulation [2, 33, 35, 50, 53, 54, 56, 58, 65, 67, 71, 75, 76, 87–94]. The other 50 % of the studies on IL-1β plasma levels in AD show slightly increased values in patients, which are yet not statistically significant due to high interindividual variances and overlaps between patients and controls. Furthermore, no study reports downregulation of IL-1β. Similar to TNF-α, it can be hypothesized that IL-1β is only elevated in subgroups of patients or during certain disease stages. Also, peripheral IL-1β might increase slowly during the time course of the disease. However, even if one of these hypotheses is correct, the effects visible in the periphery are probably small, as reflected by the large number of studies showing no significant changes between AD patients and controls. Therefore, it would be interesting to follow IL-1β-levels in AD patients’ blood and CSF longitudinally.

### IL-6

IL-6 has been examined in AD with similar frequency as TNF-α, and with similar contradictory results [2, 28, 31, 34, 52–54, 58, 60, 61, 65, 67–69, 71, 75–77, 79, 87–89, 91, 92, 95–111]. We focused on articles reporting absolute concentrations in collectives of at least 20 individuals. Both criteria were fulfilled by 18 publications [28, 30, 31, 34, 52, 58, 68, 75, 79, 91, 94, 95, 102, 103, 107, 108, 112]. Most of the studies show either upregulation or no regulation of IL-6 in blood or CSF derived from AD patients. Noteworthy is that only 2 of 18 studies report downregulation of IL-6 [75, 113]. These findings are similar between blood and CSF. Only one study analyzed IL-6 levels in the blood of MCI patients but reports no regulation [52].

When comparing the data, we made two observations which might explain the conflicts: First, all included articles showed large interindividual variances of IL-6 levels, sometimes ranging from 50–100 % of the reported mean values. As a consequence, there is a high probability that comparisons in small patient cohorts produce misleading data, as it is highly probable that some individuals will show higher or lower cytokine levels than others just by chance.

Further, patients with severe AD showed higher plasma levels of IL-6 than patients with less severe disease or healthy controls. This could be interpreted in the way that peripheral levels of IL-6 slightly increase over the time course of AD, as shown by Kalman et al [31].

These observations much resemble those made for IL-1β and TNF-α, and as before, intraindividual data over the time course of disease would be the most promising way to obtain a clearer picture regarding IL-6 levels.

### IL-6 Receptors

Levels of soluble IL-6 receptor (sIL-6R) have been analyzed in seven of the reviewed articles which investigated AD patients, but not in MCI cases [75, 81, 101, 105, 109, 112, 114]. Each of these articles report either no regulation or downregulation of sIL-6R in blood or CSF of AD patients. Absolute values are relatively consistent between the studies, ranging from approx. 20–220 ng/ml in serum and 0.5–1.6 ng/ml in CSF. Similar to the cytokines described above, high interindividual variances and a large overlap between controls and patients were observed in all studies on sIL-6R. Although the tendency to reduction of sIL-6R levels in AD is apparently weak, none of the reviewed studies reported upregulation of this cytokine receptor. This is especially interesting as IL-6 levels appear to increase slightly during AD. To our knowledge, no study so far has analyzed the ratio of IL-6 to sIL-6R in AD or changes of this ratio over the time course of disease. Again, it seems also possible that so far only uncharacterized subgroups of AD patients display lower sIL-6R levels compared to others.

### IL-18

So far, all cytokines described in this review appear to increase slowly with disease progression, while the respective receptors might be decreased. Nevertheless, some cytokines present a different picture. IL-18 has mostly been investigated in the plasma and with at first glance contradictory findings: several studies report no significant changes in IL-18 blood levels of both MCI- and AD patients, although always with a tendency to elevated levels [71, 115–117]. Two other studies show elevation of blood levels in AD [118, 119]. Most of these studies differed in the used ELISA kit and/or in patient cohort characterization, which might be one reason for the observed differences. Yet, there may be another possibility: In a study of Motta et al., the patient cohort was divided according to MMSE into mild, modest, and severe AD subgroups. These authors showed that IL-18 levels were elevated in the early stages of the disease, but later dropped again to levels equal to those of controls [27]. After the initial rise, the following decline of IL-18 levels occurred in a disease progression-dependent manner. In other words, IL-18 levels reached a peak in mild AD patients and correlated positively with the MMSE afterwards. These findings would fit to several other studies (e.g., [117, 119]) and support the concept of analyzing AD subgroups. They also support the theory of neuroinflammation as an early event in AD [120]. In this context, it is interesting to note that no study analyzing IL-18 reports effects in the plasma of MCI patients [71, 116, 117]. Together with the results of Motta et al., these findings may indicate that IL-18 levels are elevated in the early phases of AD, possibly during the turnover from “normal” MCI to AD. To our knowledge, only one study analyzed IL-18 levels in CSF of AD patients and found elevated levels of this cytokine [115]. It should further be mentioned that IL-18-binding protein (IL-18BP), a regulator of IL-18 function, has been described as downregulated in AD, indicating that the ratio of IL-18 and IL-18BP is influenced by regulation of both proteins [119]. Summarized, IL-18—and possibly its regulator IL-18BP—represent interesting candidates to be analyzed in plasma and especially CSF of well-characterized MCI and AD patients.

### CCL2/MCP-1

MCP-1 has been analyzed in plasma and CSF of AD and MCI patients. Although results were again controversial, several studies find MCP-1 to be upregulated in the CSF of AD and also MCI patients [121–123]. In plasma, most articles report no regulation of MCP-1 [51, 70, 116, 121]. Only one study conducted by Galimberti et al. investigated patients divided in MCI, mild-modest AD and severe AD groups and revealed elevated levels in MCI and mild-modest AD patients, while subjects with severe AD showed lower levels [29]. The effect strength was statistically significant, yet modest in size and there were large overlaps between the groups. However, MCP-1 levels correlated to MMSE after onset of MCI. This induction pattern is highly similar to the one described by Motta et al. for other cytokines and might be the result of innate immune activation in the early stages of AD, as mirrored by central and peripheral cytokine levels [27, 124].

### CXCL10/IP-10

The 10-kDa interferon gamma-induced protein (IP-10) is reported to be elevated in the CSF of MCI patients [82, 122]. After conversion from MCI to AD, CSF levels drop again and correlate over the time course of disease with MMSE scores and cognitive decline [82, 122, 123]. In contrast, plasma levels are uniformly reported to be unchanged in AD [70, 116, 121]. Therefore, IP-10 might resemble MCP-1 or IL-18 by showing a peak of CSF levels only in early disease stages.

### TGF-β

One of the cytokines showing the most inconsistent data is TGF-β [27, 61, 78, 85, 94, 118, 125–131]. It has been primarily investigated in AD and is described as not regulated, upregulated, downregulated and regulated dependent on disease state. We focused on eight articles which used ELISA for detection, but still found high variances in patient characterization and results [27, 78, 94, 127–131]. The mean values for healthy controls ranged from 10 pg/ml to 60 ng/ml, most likely derived from the lack of technical standardization. Still, as mentioned above, this does not explain the different directions of regulation between the reports. In contrast to IL-18, it was not possible to explain these different results based on a disease progression-dependent regulation of TGF-β.

### Cytokines with No or Marginal Changes in AD

Several cytokines have been intensively investigated in AD patients without finding an induction or regulation in blood or CSF. A good representative for this group is interleukin-2 (IL-2), which was analyzed in three studies on CSF and seven studies on plasma of AD patients [2, 33, 54, 67, 68, 71, 79, 89]. As all of these studies uniformly reported no changes in CSF or plasma levels compared to controls, IL-2 is probably not regulated in AD. Similar findings have also been documented for its receptor IL-2R and some other cytokines like GM-CSF, IFN-γ, IL-1α, IL-1RA, and IL-3 (Supplementary 2). Still, some of these factors have barely been investigated in the CSF of AD or MCI patients and it cannot be excluded that changes might be visible in CSF which are undetectable in peripheral blood.

### Other Inflammation Associated Proteins

Together with cytokines, several other proteins induced by cytokines or otherwise involved in or associated with inflammatory processes, like growth factors, selectins or acute phase proteins have been investigated (Supplementary 2). The resulting findings were often as contradictory as for cytokines, although available data might sometimes be too scarce for final conclusions. Two frequently analyzed examples are alpha-1-antichymotrypsin (ACT) and brain-derived neurotrophic factor (BDNF):

- ACT has been extensively studied in AD patients using the methods of immunodiffusion and ELISA [30, 65, 71, 72, 87, 88, 95–100, 132–135]. Data on ACT levels in MCI, on the other hand, are scarce. Approximately 50 % of the articles on ACT describe modest upregulation in AD, while the other half does not find differences in serum or CSF. It has been stated that ACT levels might show a weak positive correlation with disease progression in AD, which might explain the differences between the reports [95]. When evaluated as a biomarker, ACT levels were insufficient to discriminate AD from other dementias, whereas elevated levels in other diseases lead to a high false-positive rate [132–134].
- The effects reported for BDNF were mostly modest whereas interindividual differences were high and overlapping between the groups [35–37, 78, 136–139]. The largest study by O´Bryant et al. investigating nearly 100 individuals showed no differences between AD patients and controls [140]. Therefore, smaller collectives might provide misleading results due to the high interindividual variances, and BDNF levels might in reality be unchanged in AD.

## Conclusions

Studies on proteins involved in immune signaling and regulation often present a heterogeneous picture. Methodical variances caused by use of different ELISA kits, might be one contributing factor to the observed discrepancies. Despite from various diluents and detection methods, capture or detection antibodies might recognize different antigens, resulting in the quantification of various protein isoforms. Comparative studies between numerous antibody-based single- and multiplex approaches for cytokine quantification and a better characterization of the epitopes recognized by the respective antibodies might therefore be desirable. As recently pointed out, use of serum or plasma biobanking conditions and sample handling may significantly affect the results of cytokine detection, which is why improvement of standardization between research groups should also be considered [141].

**Fig. 1:**
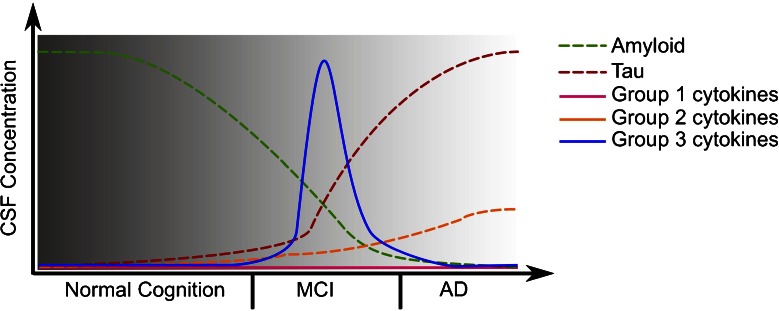
Hypothetical time course of CSF cytokine expression in AD. Graphs display the estimated CSF concentration changes of amyloid and tau protein during the development of AD, as described by others [142]. As different cytokines and other inflammatory proteins appear to display different changes in CSF levels during disease development, they might be divided into groups: First, cytokines like IL-1α or IL-2 which might remain unchanged in AD; Second, cytokines like IL-1β, IL-6 or TNF-α which might increase slowly during disease progression; third, cytokines like IL-18, MCP-1 or IP-10 which might show a peak at certain disease stages, especially at time of MCI to AD conversion. However, data becomes scarce for early disease stages. To test this hypothesis and the grouping of cytokines, longitudinal CSF sampling from individuals at risk of dementia over years would be the most efficient way

Further differences might be based on patient collective characterization, especially in terms of disease progression, as several studies discuss correlations of cytokine expression to disease state [27, 29–32, 36–38, 47]. As a possible guideline for future studies, levels of cytokines, other immune signaling related regulators and their receptors in blood or CSF of MCI and AD patients can be divided into five groups by involvement into disease, available information and consequences for research (Fig. 1):

- The first group contains cytokines like IL-2 or IL-1-α which are frequently and uniformly reported as unchanged during disease progression, especially in regard of blood levels. Of note, this does not exclude any intra- and intercellular function of these cytokines, but makes them less promising targets for biomarker research.
- The second group includes cytokines like IL-1β, IL-6, and TNF-α which seem to increase slightly but steadily over the time during the course of AD, not only in the CSF but also in blood. Members of this group often show effects which are too small to be used as reliable biomarkers. Aside from steady increase, there are the possibilities that individuals with elevated levels of these cytokines are at higher risk to develop AD or that subgroups of AD patients display elevated levels.
- The third group includes cytokines for which a peak in mild AD or around the conversion from MCI to AD has been documented. A longitudinal validation of these observations seems to be a promising target for biomarker research. Likewise, cytokines from the second group may be successfully attributed to a distinct time point of disease and thus allow for further functional insight.
- The fourth group comprises the less frequently analyzed cytokines and cytokine receptors, like CD40, which were only investigated in a limited amount of studies and require further validation. Studies of such cytokines, especially from CSF samples, could be a useful addition to the large number of already existing analyses.
- The last group includes cytokines like TGF-β, for which the documented data are just too inconsistent to allow for any interpretation. For the latter, it would helpful to optimize the characterization of the patient collective and to standardize the detection methods. When picking candidates from these groups, it should be noted that pairs of cytokines and the respective receptors or binding partners (like TNF-α and TNF receptor, IL-6 and IL-6 receptor or IL-18 and IL-18BP) often showed coregulation or inverse regulation. This observation could be useful to create ratios between cytokines and their receptors or binding partners. Such ratios could represent more valid and reliable biomarkers than each cytokine level alone.

Overall, there is a substantial lack of longitudinal data of cytokine expression, which might account for much of the contradictory results. Studies analyzing large cohorts of elderly and MCI patients over several years are missing to date. Ideally, such studies would continuously collect blood and CSF samples according to a predefined schedule. At the same time, clinical evaluations and cerebral imaging, along with the detection of the classical CSF biomarkers, amyloid, tau and phospho-tau, should be assessed and related to inflammatory mediators. Once such studies are performed, they will provide important information and allow for a more solid picture of the role of cytokine expression during AD development.

## Electronic Supplementary Material

Below is the link to the electronic supplementary material.

ESM 1 (DOCX 266 kb)

ESM 2 (DOCX 72 kb)
