# Supplementary material for: Body Fluid Cytokine Levels in Mild Cognitive Impairment and Alzheimer’s Disease: a Comparative Overview
Source: Mol Neurobiol. 2014 Feb 25;50(2):534–44. doi: 10.1007/s12035-014-8657-1 (PMC4182618; doi:10.1007/s12035-014-8657-1)
Supplement: Supplementary file 1 — (DOCX 266 kb) [file 12035_2014_8657_MOESM1_ESM.docx]

**Supplementary 1: Article Reference List**

This table gives a brief description of the 118 articles reviewed with a focus on cytokine or other inflammation associated protein levels in plasma / serum or CSF of patients with MCI and AD. Each article is assigned a list number corresponding to the supplementary reference list (see below), first author and publication year, average case number of human study participants, reported proteins and the method used for quantification. Note that multiplex assays often contain a large number of analytes not included in this review. See supplementary 2 for a protein-specific overview of results.

| **List Nr.** | **First Author** | **Year** | **Description** | **Average n** | **Reported Proteins** | **Method** |
| --- | --- | --- | --- | --- | --- | --- |
| [**1**](#_ENREF_1) | Ray S | 2007 | Quantification of 120 proteins in plasma samples of 259 individuals with presymptomatic to late-stage AD and controls. 18 signaling proteins were chosen for classification of AD vs. control samples with 90% accuracy and for identification of patients with MCI to AD turnover. Results were evaluated with partial confirmation by source 6, 38 and 117. | 58 | ANG-2, CCL5, CCL7, CCL15, CCL18, IL-8, EGF, G-CSF, GDNF, ICAM-1, IGFBP-6, IL-1a, IL-3, IL-11, M-CSF, PDGF-BB, TNF-α, TRAIL-R4 | Cytokine Array |
| [**2**](#_ENREF_2) | Westin K | 2012 | Quantification of CCL2, CCL11, CCL13 and CCL26 in CSF and plasma, as well as determination of standard CSF Tau and Aβ species, in 119 MCI patients and 30 controls. CCL26 was significantly elevated in patients with MCI to AD turnover (prodromal AD). CCL2 levels at baseline correlated with faster cognitive decline in prodromal AD patients. | 75 | CCL2, CCL11, CCL13, CCL26 | ELISA |
| [**3**](#_ENREF_3) | Mocali A | 2004 | Quantification of TGF-β1 und soluble CD40 in Plasma of 33 late onset AD patients, 40 age-matched and 11 young healthy controls, as well as PD and other dementia patients. Increase of sCD40 together with decrease of TGF-β1 was observed in AD patients, but not in other dementias compared to control groups. | 28 | sCD40, TGF-β1 | WB |
| [**4**](#_ENREF_4) | Llano DA | 2012 | Quantification of IL-1β, IL-2, IL-6, IL-8, IL-10, IL-12, GM‑CSF, IFN-γ and TNF-α over a time course of 24 h in plasma and, using lumbar catheter, in CSF of 15 AD patients and controls. No significant differences were found in cytokine levels between the groups. Yet, probably as reaction to the lumbar catheter, dynamics of the cytokines IL-1β, IL-2, IL-10, IL-12, GM-CSF, IFN-γ and TNF-α differed between AD and control group, indicating a different inflammatory response to the catheter. No differences in cytokine dynamics were observed in plasma. | 15 | IL-1b, IL-2, IL-6, IL-8, IL-10, IL-12, GM‑CSF, IFN-γ, TNF-α | ELISA |
| [**5**](#_ENREF_5) | Ait-ghezala G | 2008 | Quantification of sCD40, CD40L, and Aβ1-40 in Plasma of APOE-characterized AD patients (n=73) and controls (n=102). Increased levels were observed and declining MMSE scores correlated with increase of CD40L. In combination with APOE genotype, a specificity for AD diagnosis of >90% was reached. | 88 | sCD40, sCD40L | ELISA |
| [**6**](#_ENREF_6) | Björkqvist M | 2012 | Evaluation of the biomarker panel suggested in source 1 (Ray 2007) using comparable methods. The study included 174 healthy controls, 29 depression patients, 142 AD cases, 22 frontotemporal dementia (FTD) cases, 18 Vascular dementia (VaD) cases, 11 cases of Parkinson´s disease (PD) with dementia and 37 cases of dementia with Lewy bodies (DLB). Of the 18 cytokines suggested in source 1, only EGF, PDGF-BB und CCL15 were significantly different between controls and AD cases. Diagnostic precision of the biomarker panel in general was low and could not distinguish AD from other dementias. | 62 | ANG-2, CCL5, CCL7, CCL15, CCL18, IL-8, EGF, G-CSF, GDNF, ICAM-1, IGFBP-6, IL-1α, IL-3, IL-11, M-CSF, PDGF-BB, TNF-α, TRAIL-R4 | Cytokine Array, ELISA |
| [**7**](#_ENREF_7) | Ojala J | 2009 | Levels of IL-18 were found increased in the brain and CSF, but not in plasma of AD patients (n=49) compared to patients with Vascular dementia (VD) (n=7) and controls (n=13). IL-18 was found in Aß-plaque associated glia, blood vessels and neurons. | 23 | IL-18 | ELISA, WB |
| [**8**](#_ENREF_8) | Licastro F | 2000 | Levels of IL-1β, IL-6, and ACT were found higher in plasma of patients with AD (n=145) than in controls (n=51). No differences were observed in plasma neopterin and CRP levels. Plasma levels of ACT correlated with cognitive performance. | 98 | IL-1β, IL-6, CRP, ACT | ELISA, Immunodiffusion |
| [**9**](#_ENREF_9) | Magaki S | 2007 | There were no significant differences in the plasma levels of IL-8 between elderly controls (n = 13), MCI subjects (n = 15) and mild AD subjects (n = 7). | 18 | IL-8 | Cytokine Array |
| [**10**](#_ENREF_10) | De Luigi A | 2001 | Evaluates the presence and the source of pro- and anti- inflammatory cytokines in plasma of patients with AD (n=44), multi-infarct dementia (MID, n=7), mixed Alzheimer´s and vascular dementia (MIX, n=10), uncertain dementias (n=18) and non-demented elderly people as controls (n=43). TNF-α and sTNF-RI plasma levels were higher in MID compared to controls, whereas no differences were observed in other groups. TNF-α and IL-10 release upon stimulation with LPS was lower in AD patients. Other investigated cytokines were unchanged. | 24 | TNF-α, IL-1β, IL-6, IL-10, IL-1RA, sTNF-RI | ELISA |
| [**11**](#_ENREF_11) | Kim SM | 2011 | Quantification of IL-8, IL-10, MCP-1 and TNF-α in plasma samples from 18 AD, 20 MCI and 21 control cases. The plasma IL-8 level was lower in MCI and AD patients compared to the control group. Levels of TNF-α were similar between MCI and AD patients, but significantly lower in AD cases compared to controls. In MCI, TNF-α levels ranged between control and AD levels but did not reach significance. IL-10 and MCP-1 did not differ between the groups. | 20 | IL-8, IL-10, MCP-1, TNF-α | ELISA |
| [**12**](#_ENREF_12) | Bermejo P | 2008 | Quantification of plasma levels of IL-6, TNF-α and IFN-α in patients with AD (n=45), MCI (n=34) and healthy controls (n=28). Levels of IL-6 and TNF-1 were higher in AD compared to control group, but did not differ significantly from controls in MCI. IFN-α was higher in MCI cases compared to controls and AD patients. Levels of TNF-α were higher in MCI patients than in control group, but the differences were not statistically significant. | 36 | IL-6, TNF-α, IFN-α | ELISA |
| [**13**](#_ENREF_13) | Motta M | 2007 | The levels of IL-12, IL-16, IL-18 and TGF-β1 resulted higher in mild AD patients (n=18) and were slightly lower in moderate AD patients(n=22), whereas no significant difference was observed between severe AD patients (n=11) and non-demented age-matched controls (n=20). MMSE correlated positive with cytokine levels. Suggests that inflammation in AD is disease progress and disease‑state dependent. | 18 | IL-12, IL-16, IL-18, TGF-β1 | ELISA |
| [**14**](#_ENREF_14) | Zuliani G | 2007 | Plasma IL-1β was higher in vascular dementia (VD, n=80), AD (n=60), and cerebrovascular disease non demented (CDND, n=40) patients compared with controls (n=42). TNF‑α was higher in VD and AD compared to controls, and in VD compared to AD. IL-6 was higher in VD compared with AD. No differences were found in IL-10 levels between the groups. | 56 | IL-6, TNF-α, IL-1b, IL-10 | ELISA |
| [**15**](#_ENREF_15) | Corsi MM | 2011 | Quantification of EGF, IL-1α, Il-1β, IL-2, IL-6, IL-8, IFN-γ, MCP-1, TNF-α, VEGF, E-selectin, L-selectin and P-selectin in plasma of AD patients (n=72) and controls (n=6). Levels of IL-8, IFN-γ, MCP-1 and VEGF were significantly increased in AD. Levels of P- and L-selectin were lower in AD. Levels of other cytokines were unchanged. | 39 | EGF, IL-1α, Il-1b, IL-2, IL-6, IL-8, IFN-γ, MCP-1, TNF-α, VEGF, E-selectin, L‑selectin, P-selectin | Cytokine Array |
| [**16**](#_ENREF_16) | Galimberti D | 2003 | Quantification of IP-10, IL-8 and MCP-1 in CSF and serum of AD patients (n=21) and healthy controls. All three cytokines were increased in CSF of AD patients. No differences were found in serum samples between the groups. | 21 | IP-10, IL-8, MCP-1 | ELISA |
| [**17**](#_ENREF_17) | Lee KS | 2008 | States that measuring single cytokines to monitor CNS inflammation is insufficient due to the complexity of cytokine interactions and effects. In conclusion, 23 cytokines were quantified in plasma of patients with AD (n=10), MCI (n=25) and controls (n=19). Significant differences were observed in levels of SCF, MIF, MIG und CTACK. SCF, CTACK and MIG were elevated in AD compared to MCI and controls. MIF was elevated in MCI and AD. | 15 | IFN-α2, IL-1α, IL-2Ra, IL-3, IL-12, IL‑16, IL-18, CTACK, GRO-a, HGF, ICAM-1, LIF, MCP-3, M-CSF, MIF, MIG, ß-NGF, SCF, SCGF-β, SDF-1α, TRAIL, VCAM-1, MCP-1, IP-10 | ELISA |
| [**18**](#_ENREF_18) | Galimberti D | 2006 | Evaluation of IP-10, MCP-1 and IL-8 in CSF of AD (n=36) and MCI (n=38) patients and controls (n=41). All three cytokines were increased in patients with AD and MCI compared to controls, with a peak in MCI, and correlated positively with MMSE score after onset of dementia. MCP-1 and IL-8 did also positively correlate with age. States that inflammation might be an early event in AD pathogenesis with the highest effect in MCI and mild AD. | 36 | IP-10, IL-8, MCP-1 | ELISA |
| [**19**](#_ENREF_19) | Singh VK | 1997 | Measured plasma levels of IL-6, IL-12, IFN-a and IFN-γ in patients with AD (n=22) and controls (n=17). The level of IL‑6, but not other cytokines, was significantly elevated in the AD group. | 20 | IL-6, IL-12, IFN-α, IFN-γ | ELISA |
| [**20**](#_ENREF_20) | Richartz E | 2004 | Determination of IL-1β, IL-6, TNF-α, IFN-γ, sIL-2r, sIL-6 and sTNF-R in serum and CSF and IL-6, IL-12, TNF-α, IFN-γ, IL-5 and IL-13 in stimulated blood cell cultures of patients with AD (n=20) and controls (n=21). IL-2 in CSF and serum, as well as IL-1β and TNF-α in serum could not be detected. Significantly decreased serum levels of IL-6, and CSF levels of TNF-α were observed, whereas other cytokines were insignificantly decreased. In stimulated blood cells, decreased production of cytokines in AD patients was observed. | 21 | IL-1β, IL-6, TNF-α, IFN-γ, sIL-2r, IL-5, Il-12, Il-13, sIL-6, sTNF-R | ELISA |
| [**21**](#_ENREF_21) | Kassner SS | 2008 | Investigation of expression for inflammatory proteins by PBMC´s of AD-patients (n=5) and controls (n=5). Additionally, Plasma samples were analyzed for TNF-α and oxLDL. PBMC´s positive for TNF-α, COX-2, PARP-1, CD38, C99 and presenilin-1 were significantly increased in AD patients. Also, plasma levels of oxLDL and TNF-α were increased and correlated to PBMC-markers. | 5 | TNF-α | ELISA |
| [**22**](#_ENREF_22) | Laske C | 2008 | Quantification of SDF-1α / CXCL12 in plasma and CSF of 30 AD patients and 30 controls. A decrease was found in AD patients. | 30 | SDF-1α | ELISA |
| [**23**](#_ENREF_23) | O´Bryant SE | 2009 | BDNF serum levels were measured in AD patients and controls (n=98). No significant differences were observed in BDNF levels, and there was no correlation between levels and AD severity or cognitive functioning. | 98 | BDNF | Multiplex Assay |
| [**24**](#_ENREF_24) | Reale M | 2012 | Measurement of plasma levels of IL-18, IL-18BP, CCL5, MCP-1 and ICAM-1 in AD-patients (n=38) and controls (n=39). The ratio of IL18 / IL18-BP was altered, as were levels of other investigated cytokines in dependence of the APOE genotype and plasma Aß levels. | 39 | IL-18, IL-18BP, CCL5, MCP-1, ICAM-1 | ELISA |
| [**25**](#_ENREF_25) | Baranowska-Bik A | 2008 | Investigation of levels of Aβ1‑42 and cytokines IL-6, IL-10, TNF-α in plasma of female AD patients with early AD (n=28), moderate to severe AD (n=29) and healthy controls (n=67). Plasma Aβ1‑42 was lower only in the group of moderate to severe AD. IL-6 was significantly elevated in patients with AD compared to controls. The same trend was observable for IL-10 and TNF-α, but was not significant. No correlations between Aβ1-42 levels and cytokines were observed. | 62 | IL-6, IL-10, TNF-α | ELISA |
| [**26**](#_ENREF_26) | Alvarez XA | 1996 | Investigation of TNF-α, IL-1β and histamine in plasma of AD patients and controls (n=20). IL-1β was increased, TNF-α decreased in AD group. Notes that correlations between age and cytokine levels were different or opposite in AD patients and controls. | 20 | TNF-α, IL-1β | Radioimmunoassay |
| [**27**](#_ENREF_27) | Blum-Degen D | 1995 | Determination of IL-1β, IL-2 and IL-6 in CSF and plasma of 11 AD, 12 PD patients and 12 controls. Il-1β and IL-6 were elevated in CSF and plasma of PD and AD patients. No differences were found in levels of IL-2. | 12 | IL-1β, IL-2, IL-6 | ELISA |
| [**28**](#_ENREF_28) | Bruunsgaard H | 1999 | Measurement of TNF-α in centenarians with mild (n=31) to severe (n=40) dementia and age-matched controls (n=54), as well as younger participants. Demented centenarians had higher TNF-α levels then cognitively normal, equally-aged controls. TNF-α, IL-6, and sTNF-R2 also increased significantly with age and progression of atherosclerosis. | 42 | TNF-α | ELISA |
| [**29**](#_ENREF_29) | Tarkowski E | 1999 | Measurement of TNF-α, IL-1β and IL-6 in patients with AD, VAD and controls. Also, release of Bcl-2 from isolated human neuroblastoma cells after stimulus with TNF-α was investigated. TNF-α was found increased in serum and CSF of AD and VAD patients. IL-6 levels were indifferent in CSF, but increased in serum in both patient groups. Il-1β levels did not differ between the groups | 42 | IL-1β, IL-6, TNF-α | ELISA |
| [**30**](#_ENREF_30) | Maes M | 1999 | Analysis of plasma samples from AD patients and controls (n=15). Investigations included the cytokines IL-6 and TNF‑α. Levels of both cytokines correlated positively with age. IL-6 was elevated in patients, TNF-α decreased. | 15 | Il-6, TNF-α | ELISA |
| [**31**](#_ENREF_31) | Angelis P | 1998 | Determination of IL-6 and sIL-6R in serum of 41 AD patients and 32 controls. No differences were found in levels of IL-6, while sIL-6R was slightly decreased in AD patients. | 37 | IL-6, sIL-6R | ELISA |
| [**32**](#_ENREF_32) | Chao CC | 1994 | Determination of IL-6, TNF-α and TGF-β in serum of AD patients and controls (n=22). IL-6 and TNF-α were unchanged, while a modest elevation of TGF-β in AD group was observed. | 22 | IL-6, TNF-α, TGF-β | Cell-based bioassay |
| [**33**](#_ENREF_33) | van Duijn CM | 1990 | Determination of IL-6 in 97 AD patients and 79 controls. No differences in IL-6 levels were observed. | 88 | IL-6 | Cell-based bioassay |
| [**34**](#_ENREF_34) | Chen R | 2012 | Determination of serum levels of IL-1β and TNF-α in 43 AD-patients and 22 controls. TNF-α serum concentrations did not differ between AD patients and controls, but was elevated in patients with daytime sleepiness. Il-1β could not be detected in most samples. | 33 | TNF-α | ELISA |
| [**35**](#_ENREF_35) | Juraskova B | 2010 | Quantitation of serum TGF-β and Endoglin levels in groups of young healthy (n=63), healthy elderly (n=58) and demented elderly (n=129). TGF-β was not age-dependent, but was lower in demented patients. Endoglin levels were highest in demented elderly, and increased with ageing. | 83 | TGF-β | ELISA |
| [**36**](#_ENREF_36) | Kester MI | 2011 | Measured mRNA of CSF, ICAM, IL-8, TNF-α and CCL5 isolated from peripheral blood differed between AD patients and controls (n=23). CCL5 was the only single marker that was significantly lower expressed in AD patients. | 23 | CCL5, ICAM, IGFBP, IL-1α, IL-3, IL-8, PDGFB, TNF-α | qRT-PCR |
| [**37**](#_ENREF_37) | Forlenza OV | 2009 | Analysis of serum IL-1β levels and APOE genotype in patients with mild- to moderate AD (n=58), MCI (n=74) and age matched controls (n=31). IL-1β was elevated in MCI and AD and correlated negatively with cognitive abilities. | 87 | IL-1β | ELISA |
| [**38**](#_ENREF_38) | Marksteiner J | 2011 | 16 of the cytokines published by source 01 (Ray 2007) as effected signaling proteins in AD were evaluated in plasma of 44 patients with MCI, 96 AD patients, 26 patients with depression and cognitive impairment and 16 patients with depression, but without cognitive impairment and 19 controls. AD patients showed increased levels of EGF, GDNF, CCL15 and CCL5, MCI patients increased levels of CCL18and CCL5. Yet, the overall expression pattern of proteins could not fulfill sensitivity and specificity criteria for biomarkers. | 46 | ANG-2, CCL5, CCL7, CCL15, CCL18, IL-8, EGF, G-CSF, GDNF, ICAM-1, IGFBP-6, IL-1α, IL-3, IL-11, M-CSF, PDGF-BB, TNF-α | Cytokine Array |
| [**39**](#_ENREF_39) | Hernanz A | 2007 | Investigation of levels of sRAGE, TNF-αR2, glutathione, cysteinglycine, cysteine and homocysteine in patients with AD (n=25), MCI (n=26), and controls (n=44). sRAGE and TNF-αR2 levels were unchanged. Glutathione and cysteineglycine were decreased in AD and MCI patients. Homocysteine levels were also increased and highest in AD. | 32 | TNF-αR2 | ELISA |
| [**40**](#_ENREF_40) | Rota E | 2006 | Analysis of IL-12, IFN-γ, IL-10 and TGF-β1 in CSF and serum of patients with AD (n=30), controls (n=25) and other dementia (19 VAD, 14 normal pressure hydroyephalus (NPH), 10 PD with dementia, 14 PD without dementia). TGF‑β1, but not IL-12, IFN-gamma or IL-10 were increased in AD patients compared other groups. | 19 | IL-12, IL-10, TGF-β1 | ELISA |
| [**41**](#_ENREF_41) | Alvarez A | 2007 | Determination of serum TNF-α and IGF-I levels in 141 AD patients, 56 MCI patients and 30 controls. TNF-α and IGF-I both correlated with age. In MCI and AD patients, levels of TNF-α were higher and levels of IGF-I were lower than in controls. | 76 | TNF-α, IGF-I | ELISA |
| [**42**](#_ENREF_42) | Galimberti D | 2006 | Determination of serum levels of MCP-1 in patients with MCI (n=48), AD (n=94, divided in mild-modest and severe AD) and controls (n=24). MCP-1 was increased in patients, with highest levels in MCI and mild-modest AD, while severe AD patients showed lower levels. MCP-1 levels correlated positively with MMSE after onset of MCI. | 55 | MCP-1 | ELISA |
| [**43**](#_ENREF_43) | Lindberg C | 2005 | Quantification of serum and CSF sIL-1R2, IL-18 and caspase-1 of MCI patients which later tuned to AD (n=20), severe AD patients (n=12) and controls (n=11). CSF IL-18 and caspase-1 could not be detected. Other CSF and serum levels were unchanged. | 14 | sIL-1R2, IL-18 | ELISA |
| [**44**](#_ENREF_44) | Solerte SB | 2000 | Isolation of NK immune cells from AD patients (n=22) and controls (n=15) for determination of both spontaneous and IL-2 stimulated release of TNF-α and IFN-γ. Serum levels of both cytokines were equal between groups, whereas NK cells of AD patients showed increased release of cytokines after stimulus. | 19 | TNF-α, IFN-γ | ELISA |
| [**45**](#_ENREF_45) | O´Bryant SE | 2010 | CRP determined in serum of 192 AD patients and 174 controls was lower in AD cases, although higher CRP levels were associated with faster cognitive decline in AD patients. | 183 | CRP | Multiplex Assay |
| [**46**](#_ENREF_46) | Hasegawa Y | 2000 | Determination of IL-1α, IL-1β, IL-6, M-CSF, G-CSF, TNF-α, IL-1RA, sIL-6R and sTNF-R in 25 AD patients and controls aged 74-96 (elderly, n=24), 50-59 (middle-aged, n=12) and 22-38 (younger). M-CSF increased with age and had highest levels in AD patients. IL-1α, IL-1RA, IL-1β, TNF-α and IL-6 could not be detected. G-CSF, sIL-6R did not differ between groups. sTNF-R increased with aged, but did not differ between AD and healthy elderly controls. | 20 | M-CSF, G-CSF,sIL-6R, sTNF-R | ELISA |
| [**47**](#_ENREF_47) | Tarkowski E | 2003 | Analysis of TNF-α, TGF-β, IL-1b, GM-CSF, Tau and Aß42 in patients with MCI (n=25) and AD (n=31) after a 9-month follow-up and healthy controls (n=25). TGF-β was decreased, TNF-α increased in patients with MCI and AD. GM-CSF could not be detected in an sufficient amount of participants. IL-1b did not differ between groups. | 27 | IL-1β, TNF-α, TGF-β | ELISA |
| [**48**](#_ENREF_48) | Lanzrein AS | 1998 | Longitudinal study over 2-5 years investigating the correlation between cytokines IL-1β, IL-6, Il-1RA, TNF-α, sTNF-R1, sTNF-R2 and ACT with cognitive decline in 8 AD patients and 9 controls. No differences between the groups were found, and no correlations of the investigated markers with cognitive decline. | 9 | IL-1β, IL-6, Il-1RA, TNF-α, sTNF-R1, sTNF-R2, ACT | ELISA |
| [**49**](#_ENREF_49) | Lepara O | 2009 | AD patients and controls (n=30) where compared for serum levels of CRP and homocysteine. CRP was unchanged in AD patients, while homocysteine was elevated. Both markers did not correlate. | 30 | CRP | Immunoephelometrie |
| [**50**](#_ENREF_50) | Mashayekhi F | 2010 | CSF and serum FGF-1 levels were compared between AD patients and controls (n=32). In both sample types, FGF-1 was elevated in AD patients. | 32 | FGF | ELISA, WB |
| [**51**](#_ENREF_51) | Engelborghs S | 1999 | Investigation of 42 AD-patients and matched controls revealed no significant changes in CSF levels of IL-1β, IL-6, IL-10, IL-12, sIL-2R, INF-g, TNF-α and neopterin. | 42 | IL-1β, IL-6, IL-10, IL-12, sIL-2R, IFN-γ, TNF-α | ELISA |
| [**52**](#_ENREF_52) | Fillit H | 1991 | Quantitation of serum TNF-α levels in 24 AD patients and controls with the result of significantly elevated TNF-α in AD patients. | 24 | TNF-α | Cell-based bioassay, ELISA |
| [**53**](#_ENREF_53) | Garlind A | 1999 | Quantitation of CSF sIL-1R2, IL-1RA, IL-1β, IL-6 and TNF-α levels in 12 AD patients and 13 controls with the result of significantly elevated TNF-α in AD patients. IL-1β and IL‑1RA could not be determined. sIL-1R2 was increased, while IL-6 and TNF-α showed no differences between groups. | 13 | sIL-1R2, IL-6, TNF-α | ELISA |
| [**54**](#_ENREF_54) | Craig-Schapiro R | 2011 | 190 analytes were measured in CSF of cognitively normal (n=242), very mild demented (63) and mild demented patients (n=28). In both dementia groups, VEGF and IL-7 were reduced, while MIF, MCP-2, Eotaxin-3, IL-10, TNF-RII, IP-10 and MIG were elevated. Best estimator for development of mild dementia was a combination of Aß42 and calbindin level and age. | 111 | VEGF, IL-7, MIF, Eotaxin-3, IL-10, TNF-RII, IP-10, MIG | Multiplex Assay |
| [**55**](#_ENREF_55) | Hampel H | 1997 | IL-6 was determined in CSF of 25 AD patients and 19 controls without showing significant differences. | 22 | IL-6 | ELISA |
| [**56**](#_ENREF_56) | Hampel H | 1999 | Quantitation of CSF sIL-1R2, IL-6 and Tau levels in 25 AD patients and 19 controls. No regulations were found despite of increased CSF Tau protein. Combination of Tau and sIL‑1R levels reached >90% sensitivity and specificity for discrimination of AD from controls. | 22 | IL-6, sIL-6R | ELISA |
| [**57**](#_ENREF_57) | Harigaya Y | 1995 | Levels of ACT in CSF were measured in 66 AD patients and 54 controls. ACT levels did not correlate with aging and were significantly higher in AD patients, especially in the subgroup of late onset AD compared to early onset AD and controls. | 60 | ACT | ELISA |
| [**58**](#_ENREF_58) | Licastro F | 1995 | Determination of serum levels of ACT together with other acute phase proteins in patients with AD (n=29), VAD (15) and controls (n=21). ACT was elevated in AD patients compared to controls and VAD. | 22 | ACT | ELISA |
| [**59**](#_ENREF_59) | Licastro F | 2000 | Concentration of ACT and CRP were measured in serum or plasma from 137 AD patients and 89 controls. Serum levels were higher than plasma levels. ACT was elevated in AD, whereas CRP was unchanged. | 113 | ACT, CRP | Immunodiffusion |
| [**60**](#_ENREF_60) | Martinez M | 1993 | Study of IL-1β, β2-microglobulin, β-endorphin, substance P, neuropeptide Y and somatostatin in CSF of 13 AD patients, 13 multi-infarct-dementia (MID) patients and 15 controls. Substance P and somatostatin were lower in AD patients compared to controls, whereas β2-microglobulin was enriched. No differences were found in levels of Il-1β, neuropeptide Y and β-endorphin. | 14 | IL-1β | Radioimmunoassay |
| [**61**](#_ENREF_61) | Martinez M | 2000 | Determination of soluble Fas, IL-1β and IL-6 in CSF of AD-patients and controls (n=10). IL-6 and Fas were found increased, whereas IL-1β concentrations were unchanged. | 10 | IL-1β, IL-6 | ELISA |
| [**62**](#_ENREF_62) | Marz P | 1997 | Determination of IL-6, sIL-6R and IL-6 signal transducing protein gp130 in CSF of 17 AD patients and 18 controls. No significant differences were observed. | 18 | IL-6, sIL-6R | ELISA |
| [**63**](#_ENREF_63) | Doecke JD | 2012 | A plasma biomarker panel based on 754 healthy controls and 207 AD patients was validated in 58 controls and 112 AD patients from a different cohort. The panel included increased cytokines VCAM-1, CD40, MIP-1a, and decreased cytokines EGF-R and IL-17. | 283 | VCAM-1, sCD40, MIP-1α | Multiplex Assay |
| [**64**](#_ENREF_64) | Blasko I | 2006 | Evaluation of Aβ1-42, total-tau, phospho-tau-181, HGF, GDNF, VEGF, BDNF, FGF-2, TNF-α, TGF-β and MIP-1α in CSF of 23 AD patients, 5 FTLD patients, 11 patients with depression, 10 alcohol-induced dementia cases and 27 controls. P-tau-181 / Aß1-42 ratios significantly distinguished AD cases from healthy controls and other subgroups. Growth factors and cytokines did not differ significantly between the groups. MCP-1α correlated positively with age. NGF levels correlated negatively with p-tau-181 / Aß1-42 ratio and positively with MCP-1 level. | 15 | HGF, BDNF, GDNF, VEGF, FGF2, TNF-α, TGF-β, MIP-1α | ELISA |
| [**65**](#_ENREF_65) | Pirttila T | 1994 | Quantitation of ACT and IL-1β in paired serum and CSF samples of 40 patients with AD, 20 patients with PD and 42 controls. No significant differences were found between the groups. | 34 | ACT, IL-1b | ELISA |
| [**66**](#_ENREF_66) | Yamada K | 1995 | Determination of IL-6 level in patients with AD (n=12), VD (n=8) and controls (n=7). IL-6 was significantly reduced in CSF of early-onset AD patients. No correlation between IL-6 and severity of dementia or disease duration since onset was found. | 9 | IL-6 | ELISA |
| [**67**](#_ENREF_67) | Licastro F | 1995 | Levels of ACT in CSF and serum of patients with early-onset AD and late AD, VD and controls were compared. In CSF, ACT levels were higher in AD patients compared to VD cases and controls and correlated with disease stage. No differences were found in serum | 18 | ACT | ELISA |
| [**68**](#_ENREF_68) | Hampel H | 1998 | Levels of sIL-6R were decreased in CSF of patients with AD (n=41) compared to controls (n=20). | 20 | sIL-6R | ELISA |
| [**69**](#_ENREF_69) | Angelucci F | 2010 | Serum BDNF levels were increased in MCI (n=54), mild AD (n=54) and moderate-severe AD (n=35) patients compared to controls (n=27). Levels were independent of disease progression. | 45 | BDNF | ELISA |
| [**70**](#_ENREF_70) | Li G | 2009 | CSF concentration of BDNF, Aß1-42 and total tau was measured in 128 cognitive normal adults, 21 AD patients and 9 MCI patients. CSF BDNF was lower in AD patients compared to controls and decreased with age. | 53 | BDNF | Multiplex Assay |
| [**71**](#_ENREF_71) | Angelopoulos P | 2008 | Levels of IL-1β, IL-6, IL-10, IL-2 and TNF-α were determined in serum of 56 AD patients, 16 VD patients and 15 controls. IL-1β, Il-6 and IL-10 were elevated in dementia patients samples. IL-6 and TNF-α correlated with age. | 29 | IL-1β, IL-2, IL-6, Il-10, TNF-α | ELISA |
| [**72**](#_ENREF_72) | Buchhave P | 2009 | Follow-up-study of 4-7 years after measurement of plasma sCD40 and sCD40L at baseline including 136 MCI patients and 30 controls. Sixty MCI patients converted to AD within the observation time. Converters had elevated levels of sCD40, but not sCD40L, at baseline compared to controls, and sCD40 levels correlated to cognitive decline and CSF Aβ isoforms. sCD40-levels in non-converters were similar to controls. | 83 | sCD40, sCD40L | ELISA |
| [**73**](#_ENREF_73) | Murase K | 1993 | Measurement of NGF levels in serum, CSF and post mortem material from AD patients (n=8) and controls (n=9). No differences in NGF levels were observed, but NGF negatively correlated with age. | 9 | β-NGF | ELISA |
| [**74**](#_ENREF_74) | Lieberman J | 1995 | Measurement of ACT in serum of 57 AD patients, 86 age-matched controls, 67 non-AD geriatric patients, 56 adult and 80 children asthmatics, as well as 24 healthy children. Serum ACT was elevated in AD-group compared to others. Notes that ACT still cannot used as diagnostic criterion because of 26% false positive rate with elevated levels due to other causes. | 62 | ACT | Immunodiffusion |
| [**75**](#_ENREF_75) | DeKosky ST | 2003 | ACT concentrations were determined in plasma of 359 AD subjects, 44 patients with other late-life dementias, and 113 controls. Plasma ACT levels correlated with AD group and disease severity. ACT levels in CSF of 34 AD cases and 16 controls were also elevated. Similar elevation was found in other dementias. Article also notes that ACT might not be suited as diagnostic marker, but might still reflect disease progression. | 172 | ACT | Immunodiffusion |
| [**76**](#_ENREF_76) | Han Y | 2012 | Plasma samples of 112 AD patients, 85 VD patients, 30 patients with other dementia and 116 non-demented controls were compared. ACT was elevated in AD patients compared to controls, but did not reach sufficient sensitivity and specificity to differentiate between groups. Combination with levels of amyloid isoforms increased usability as biomarker. | 86 | ACT | ELISA |
| [**77**](#_ENREF_77) | Licastro F | 2001 | Blood was collected from patients with AD and controls (n=11) three times over a time interval of up to 10 days. One sample was obtained from VAD patients (n=6). ACT, CRP and oxidative stress protein markers were determined. Erythrocyte SOD was increased in AD patients. Absolute values of ACT and were higher in AD patients and VAD patients and correlated with cognitive decline, while CRP was unchanged. | 9 | ACT, CRP | Immunodiffusion |
| [**78**](#_ENREF_78) | O´Bryant SE | 2011 | No differences in serum BDNF-levels were found in 198 AD patients and 201 controls. | 200 | BDNF | Multiplex Assay |
| [**79**](#_ENREF_79) | De Servi B | 2002 | Determination of plasma TGF-β levels and NOS activity of leukocytes in 48 sporadic AD patients and 23 controls. TGF‑β was reduced in AD group, while NOS activity was increased. | 36 | TGF-β | WB |
| [**80**](#_ENREF_80) | Dicou E | 1997 | No changes of NGF and anti-NGF-autoantibodies and no correlations of levels with age were observed in sera of AD patients and controls (n=28). | 28 | β-NGF | ELISA |
| [**81**](#_ENREF_81) | Laske C | 2006 | Investigation of serum and CSF BDNF concentration in a pilot study including early and late-stage AD patients (each n=15) and 10 controls. BDNF serum concentrations were elevated in early stage AD patients compared to other AD patients and controls. Also, BDNF serum values correlated with MMSE score. | 13 | BDNF | ELISA |
| [**82**](#_ENREF_82) | Laske C | 2007 | Investigation of serum and CSF BDNF concentration in a new cohort of late-stage AD patients (n=27), normal pressure hydrocephalus (NPH) patients (n=9) and 28 controls. BDNF serum concentrations were lower in late stage AD patients compared to controls and did not correlate with MMSE score. CSF levels were close to detection limit without showing significant differences. Authors note that BDNF levels might be disease progression dependent, as indicated by their pilot study source 81. | 21 | BDNF | ELISA |
| [**83**](#_ENREF_83) | Bonotis K | 2008 | Investigated levels of IL-1α, IL-2, IL-6, IL-8, IL-10, TNF-α and CD4 and CD8 T-lymphocytes in AD patients with mild (n=30) and severe (n=19) cognitive impairment and controls (n=21). TNF-α was increased in severe stage patients, while CD4 subpopulation was decreased. No significant differences were found in other investigated cytokines. | 23 | IL-1α, IL-2, IL-6, IL-8, IL-10, TNF-α | ELISA |
| [**84**](#_ENREF_84) | Teunissen CE | 2003 | Serum from patients with AD (n=34), PD (n=46), other dementia (n=47) and controls (n=61) was investigated for 11 cytokines, 12 sterol intermediates and phytosterols, 2 brain-specific proteins and 4 homocysteine homeostasis constituents. Best discrimination between AD patients and controls was achieved with a combination of IL-6R, protein alpha1 fraction, cysteine and cholesterol. IL-6R was lower in patients with dementia, while CRP and IL-6 were not changed. | 47 | IL-6, IL-6R, CRP | ELISA |
| [**85**](#_ENREF_85) | Laske C | 2011 | BDNF serum levels were decreased in 12 AD patients with fast cognitive decline compared to 28 AD patients with fast cognitive decline. | 20 | BDNF | ELISA |
| [**86**](#_ENREF_86) | Inspector M | 2005 | Levels of matrix metalloproteinase-9, TIMP-1 and CRP were measured in plasma of 10 patients with mild AD, 9 with moderate to severe AD, and 10 controls. No significant differences were observed between AD patients and controls. Plasma levels did not correlate to APOE genotype. Patients not treated with Acetylcholine esterase inhibitors showed significantly higher levels of TIMP-1 and CRP then untreated patients and insignificantly higher levels then controls and mild stage AD patients. | 10 | CRP | ELISA |
| [**87**](#_ENREF_87) | Zuliani G | 2008 | Plasma levels of VCAM-1, IL-1β, IL-6, TNF-α, IFN-γ, E‑selectin were investigated in 60 late-onset AD patients, 80 VD patients, 40 cerebrovascualar, but not demented patients and 30 controls. VCAM-1 was significantly higher in demented patients and insignificantly higher in cerebrovascular patients compared to controls. IL-1β was higher in all patients compared to controls. TNF-α was higher in demented patients and highest in VD patients. IL-6 was higher in VD then in AD. | 53 | VCAM-1, IL-1β, IL-6, TNF-α, IFN-γ, E‑selectin | ELISA |
| [**88**](#_ENREF_88) | Genc S | 2009 | Investigation of TRAIL serum and CSF levels and TRAIL mRNA expression in PBMC´s in 22 AD patients and 20 controls. TRAIL could not be detected in CSF and was unchanged in serum and PBMC´s. | 21 | TRAIL | ELISA |
| [**89**](#_ENREF_89) | Laske C | 2010 | In plasma of 50 AD patients, increased M-CSF levels were found compared to 22 MCI patients, 20 non-inflammatory neurological disease (NIND) patients and 35 controls. In Plasma, M‑CSF was elevated only in AD patients. MCI patients showed decreased CSF M-CSF levels compared to AD patients and NIND group. CSF M-CSF levels of AD group were similar to NIND group. | 32 | M-CSF | ELISA |
| [**90**](#_ENREF_90) | Yasutake C | 2006 | Serum levels of BDNF, TNF-α and IL-1β were investigated in 60 AD patients, 60 VD patients and 33 controls. BDNF was slightly lower in AD patients then in VD patients and controls, whereas the other markers did not differ between the groups. | 51 | BDNF, TNF-α, IL-1b | ELISA |
| [**91**](#_ENREF_91) | Choi C | 2008 | 22 Cytokines and chemokines were analyzed in serum of 13 controls, 11 AD- and 8 PD-patients. In CSF, only MCP-1 was detectable and did not differ between the groups. In serum, only eotaxin was significantly lower in AD group. | 11 | IL-1α, IL-7, IL-8, IL-12, IFN-γ, GM-CSF, Eotaxin, IP-10, MCP-1, MIP-1, CCL5, TNF-α | Multiplex Assay |
| [**92**](#_ENREF_92) | Lawlor BA | 1996 | Blood concentrations of ACT, CRP, alpha 1 antithrombin and C1 esterase inhibitor were investigated in 17 AD patients and 15 controls without finding significant differences. | 16 | ACT, CRP | Immunodiffusion |
| [**93**](#_ENREF_93) | Mulder SD | 2009 | Levels of ACT and PSA-ACT were determined in CSF and serum samples of 9 AD patients, 9 FTLD patients, 9 MCI patients and 6 controls. No differences in ACT or PSA-ACT levels were found between the groups. | 8 | ACT | ELISA |
| [**94**](#_ENREF_94) | Chao CC | 1994 | Quantitation of TGF-β levels in serum and CSF pre- and post mortem of AD patients and controls (n=6 pre-mortem and n=9 post-mortem). Postmortem serum and CSF and pre-mortem serum levels of TGF-β were increased. | 8 | TGF-β | Cell-based bioassay |
| [**95**](#_ENREF_95) | Laske C | 2009 | Significantly lower G-CSF levels were found in plasma of 50 early AD patients compared to 50 controls. G-CSF correlated with age and Aβ1-42 levels but not with MMSE or tau. | 50 | G-CSF | ELISA |
| [**96**](#_ENREF_96) | Helmy AA | 2012 | 10 AD patients, 10 VD patients and 20 controls were evaluated by MRI, serum IL-6, CRP and protein electrophoresis. In both types of dementia, levels of IL-6 and CRP were elevated. | 10 | IL-6, CRP | ELISA |
| [**97**](#_ENREF_97) | Cojocaru IM | 2011 | Serum IL-6 was investigated in 47 AD patients and 47 controls. IL-6 was higher in AD group. | 47 | IL-6 | ELISA |
| [**98**](#_ENREF_98) | Jia JP | 2005 | Tau, Aβ1-42 and the cytokines IL-2, IL-6 and TNF-α were measured in CSF in 35 controls, 39 AD and 38 VD cases. Tau, IL-6 and TNF-α were elevated in AD patients, while no significant differences were found in IL-2 levels. IL-6 and Tau were similar high in AD and VD, whereas TNF-α was even higher in VD then in AD. | 37 | IL-2, IL-6, TNF-α | ELISA |
| [**99**](#_ENREF_99) | Rosler N | 2001 | CSF of 27 AD, 24 other dementias, 70 CNS disease without dementia patients and 49 controls were investigated for levels of ApoE, Aβ1-42, IL-6, substance P and total Tau. Compared to other groups, Tau, IL-6 and ApoE were increased in AD, although large overlaps were observed for IL-6 and ApoE levels. Substance P was only increased in late-onset AD patients but equal in early-onset patients and controls. Aβ1-42 was reduced in AD group and could be used together with Tau to discriminate AD from controls and other dementia. | 43 | IL-6 | ELISA |
| [**100**](#_ENREF_100) | Gomez-Tortoza E | 2003 | Total Tau, Aβ1-42, Il-1b and Il-6 were measured in CSF of 33 AD patients, 25 DLB patients and 46 controls. Levels of IL-1β did not differ between patients. IL-6 showed elevated tendency but was not significant. | 35 | IL-1β, IL-6 | ELISA |
| [**101**](#_ENREF_101) | Galimberti D | 2008 | CSF levels of IL-6, IL-11 and LIF were measured in 43 AD, 24 FTLD patients and 30 controls and correlated to levels of Aβ1-42, total tau and phospho-tau 181. IL-11 was elevated in AD and FTLD patients, whereas IL-6 was unchanged and LIF undetectable. IL-11 levels correlated with MMSE, but not with tau or Aβ isoforms. Highest IL-11 levels were observed in patients with less severe degree of cognitive deterioration. | 33 | IL-6, IL-11 | ELISA |
| [**102**](#_ENREF_102) | Tarkowski E | 2002 | Levels of VEGF and TGF-β were increased in CSF of 20 AD patients and 26 VD patients compared to 27 controls. | 24 | VEGF, TGF-β | ELISA |
| [**103**](#_ENREF_103) | Zetterberg H | 2004 | Concentration of TGF-β was compared in AD patients and controls (n=20) and found to be increased in AD patients. Overall, TGF-β levels negatively correlated with Aβ1-42 levels. | 20 | TGF-β | ELISA |
| [**104**](#_ENREF_104) | Correa JD | 2011 | Investigation of Aß, Tau, phospho-Tau and the chemokines CCL2, IL-8 and CXCL10 in CSF of 22 AD patients and 27 controls. CCL2 was increased in AD and correlated with phospho-tau-levels. All chemokines correlated with Aß. | 25 | CCL2, IL-8, CXCL10 | ELISA |
| [**105**](#_ENREF_105) | Ozturk C | 2007 | Measured levels of IL-1β, IL-2, IL-6, IL-18, TNF-α, Aβ1-40 and ACT in plasma of patients with AD (n=29), MCI (n=13), VD (n=17) and controls (n=23). In all patients groups, cytokine levels were insignificantly elevated in patients, but only ACT in VD differed significantly from controls. | 21 | IL-1β, IL-2, IL-6, IL-18, TNF-α, ACT | ELISA |
| [**106**](#_ENREF_106) | Malaguarnera L | 2006 | Plasma levels of IL-18 and TGF-β were elevated in patients with AD (n=40) and VD (n=35) compared to controls (n=30). | 35 | IL-18, TGF-β | ELISA |
| [**107**](#_ENREF_107) | Matsubara E | 1989 | ACT levels in serum of patients with AD (n=20) were higher than in VD (n=15), mixed dementia (n=10), several other dementia and controls (n=77). Mean values were highest in AD and mixed dementia, and both types of dementia overlapped with other tested groups values. | 21 | ACT | Immunodiffusion |
| [**108**](#_ENREF_108) | Cacabelos R | 1994 | Levels of TNF-α were studied in serum of patients with AD (n=14), MID (n=14) and controls (n=12). TNF-α levels were lower in AD and MID patients compared to controls and correlated with age. | 13 | TNF-α | ELISA |
| [**109**](#_ENREF_109) | Rodriguez-Rodriguez E | 2007 | TGF-β polymorphisms were investigated in 412 AD patients and 406 controls. Serum levels were analyzed in 63 AD patients and 77 controls. No differences in polymorphism distribution or TGF-β levels in serum were found in AD cases. TGF-β levels did not correlate with polymorphisms. | 70 | TGF-β | ELISA |
| [**110**](#_ENREF_110) | De Luigi A | 2002 | Circulating cytokines and release of cytokines from PBMC´s upon inflammatory stimulus were investigated in patients with AD (41 mild AD, 17 moderate AD), multiinfarct dementia (n=12), mixed dementia (10) and controls (47). Circulating IL-1β and TNF-α was increased in mild dementia, IL-1b also in moderate AD. Peripheral plasma levels of sTNF-R and IL‑1RA did not differ between the groups. Release of TNF-α, IL‑10 and IL-6 from stimulated blood cells was reduced. Concludes that cytokine release from stimulated blood cells is down regulated in AD and multiinfarct dementia, while circulating levels are elevated. | 25 | IL-1β, TNF-α, sTNF-R, IL-1RA | ELISA |
| [**111**](#_ENREF_111) | Diniz BS | 2010 | TNF-α, sTNF-RI and sTNF-RII were investigated in serum of patients with MCI (n=72), AD (n=31) and controls (n=64). No differences between the groups were found. Still, MCI patients with high sTNF-RI levels had a higher risk to process to AD on follow-up after 10 month. | 56 | TNF-α, sTNF-RI, sTNF-RII | ELISA |
| [**112**](#_ENREF_112) | Bonaccorso S | 1998 | Plasma availability of tryptophan and cytokines IL-6 and IL-8 were investigated in AD patients (n=15) and controls (n=31). IL-6, but not IL-8, was higher in AD patients. | 23 | IL-6, IL-8 | ELISA |
| [**113**](#_ENREF_113) | Kalman J | 1997 | In patients with mild to moderate AD (n=15), severe AD (n=26), controls (n=24) and demented down syndrome patients (n=29), mostly normal serum IL-6-levels were found. Only in patients with severe disease, serum IL-6 was elevated. No differences were observed in CSF IL-6 levels. | 20 | IL-6 | ELISA |
| [**114**](#_ENREF_114) | Vom Berg J | 2012 | In APP/PS1 mouse model, increased production of IL-23 and IL-12 p40 was found, and in CSF of 39 AD patients compared to 20 controls, IL-12 p40 correlated with MMSE. | 30 | IL-12 p40 | ELISA |
| [**115**](#_ENREF_115) | Zhang R | 2013 | Investigation of blood samples from patients with early AD and controls. MCP‑1 expression in monocytes was decreased in AD, but plasma MCP‑1‑levels, macrophage activation levels and CD8+ T-lymphocytes were increased. | 36 | MCP-1 | ELISA |
| [**116**](#_ENREF_116) | Huang L | 2013 | Quantification of serum TGF‑ß and VEGF in patients with MCI, AD and controls. VEGF was decreased in both MCI and AD; TGF‑ß in AD. Both factors correlated positively with each other. | 29 | TGF-ß, VEGF | ELISA |
| [**^117^**](#_ENREF_117) | Soares HD | 2009 | Multiplex analysis of 89 analytes from plasma of 19 AD cases and 22 controls with sampling time points at baseline, 3, 6 and 12 month. Mean values of cytokines were not reported to differ between groups and appaerently independent from time point. Multianalyte analysis was performed with five chosen analytes (IL-3, RANTES, IL-8, ICAM-1 and TNF-α) in comparison to results from source 1 (Ray et al 2007) and a larger, one-time point cohort (61 AD, 952 controls). Concludes that overall sensitivity and specificity of plasma cytokine-based multi-analyte models is not better then risk factors like age or APOE genotype. | 21 (1^st^ cohort) | IL-3, IL-8, ICAM-1, RANTES, TNF-α | Multiplex Assay |
| [**^118^**](#_ENREF_118) | Doecke JD | 2012 | Multianalyte profiling study with a training set of 754 controls and 207 AD cases, validated in a set of 58 controls and 112 AD cases. An 18 biomarker panel was identified as best discriminator, including regulated cytokines. | 481 (1^st^ cohort) | IL-10, MIP-1α, sCD40, VCAM1 | Multiplex Assay |

Supplementary References List:

**1**. Ray S, Britschgi M, Herbert C, et al. Classification and prediction of clinical Alzheimer's diagnosis based on plasma signaling proteins. Nat Med 2007;13:1359-1362.

**2**. Westin K, Buchhave P, Nielsen H, Minthon L, Janciauskiene S, Hansson O. CCL2 is associated with a faster rate of cognitive decline during early stages of Alzheimer's disease. PLoS One 2012;7:e30525.

**3**. Mocali A, Cedrola S, Della Malva N, et al. Increased plasma levels of soluble CD40, together with the decrease of TGF beta 1, as possible differential markers of Alzheimer disease. Exp Gerontol 2004;39:1555-1561.

**4**. Rao JS, Kellom M, Kim HW, Rapoport SI, Reese EA. Neuroinflammation and synaptic loss. Neurochem Res 2012;37:903-910.

**5**. Ait-ghezala G, Abdullah L, Volmar CH, et al. Diagnostic utility of APOE, soluble CD40, CD40L, and Abeta1-40 levels in plasma in Alzheimer's disease. Cytokine 2008;44:283-287.

**6**. Bjorkqvist M, Ohlsson M, Minthon L, Hansson O. Evaluation of a previously suggested plasma biomarker panel to identify Alzheimer's disease. PLoS One 2012;7:e29868.

**7**. Ojala J, Alafuzoff I, Herukka SK, van Groen T, Tanila H, Pirttila T. Expression of interleukin-18 is increased in the brains of Alzheimer's disease patients. Neurobiol Aging 2009;30:198-209.

**8**. Licastro F, Pedrini S, Caputo L, et al. Increased plasma levels of interleukin-1, interleukin-6 and alpha-1-antichymotrypsin in patients with Alzheimer's disease: peripheral inflammation or signals from the brain? J Neuroimmunol 2000;103:97-102.

**9**. Magaki S, Mueller C, Dickson C, Kirsch W. Increased production of inflammatory cytokines in mild cognitive impairment. Exp Gerontol 2007;42:233-240.

**10**. De Luigi A, Fragiacomo C, Lucca U, Quadri P, Tettamanti M, Grazia De Simoni M. Inflammatory markers in Alzheimer's disease and multi-infarct dementia. Mech Ageing Dev 2001;122:1985-1995.

**11**. Kim SM, Song J, Kim S, et al. Identification of peripheral inflammatory markers between normal control and Alzheimer's disease. BMC Neurol 2011;11:51.

**12**. Bermejo P, Martin-Aragon S, Benedi J, et al. Differences of peripheral inflammatory markers between mild cognitive impairment and Alzheimer's disease. Immunol Lett 2008;117:198-202.

**13**. Motta M, Imbesi R, Di Rosa M, Stivala F, Malaguarnera L. Altered plasma cytokine levels in Alzheimer's disease: correlation with the disease progression. Immunol Lett 2007;114:46-51.

**14**. Zuliani G, Guerra G, Ranzini M, et al. High interleukin-6 plasma levels are associated with functional impairment in older patients with vascular dementia. Int J Geriatr Psychiatry 2007;22:305-311.

**15**. Corsi MM, Licastro F, Porcellini E, et al. Reduced plasma levels of P-selectin and L-selectin in a pilot study from Alzheimer disease: relationship with neuro-degeneration. Biogerontology 2011;12:451-454.

**16**. Galimberti D, Schoonenboom N, Scarpini E, Scheltens P. Chemokines in serum and cerebrospinal fluid of Alzheimer's disease patients. Ann Neurol 2003;53:547-548.

**17**. Lee KS, Chung JH, Lee KH, Shin MJ, Oh BH, Hong CH. Bioplex analysis of plasma cytokines in Alzheimer's disease and mild cognitive impairment. Immunol Lett 2008;121:105-109.

**18**. Galimberti D, Schoonenboom N, Scheltens P, et al. Intrathecal chemokine synthesis in mild cognitive impairment and Alzheimer disease. Arch Neurol 2006;63:538-543.

**19**. Singh VK, Guthikonda P. Circulating cytokines in Alzheimer's disease. J Psychiatr Res 1997;31:657-660.

**20**. Richartz E, Stransky E, Batra A, et al. Decline of immune responsiveness: a pathogenetic factor in Alzheimer's disease? J Psychiatr Res 2005;39:535-543.

**21**. Kassner SS, Bonaterra GA, Kaiser E, et al. Novel systemic markers for patients with Alzheimer disease? - a pilot study. Curr Alzheimer Res 2008;5:358-366.

**22**. Laske C, Stellos K, Eschweiler GW, Leyhe T, Gawaz M. Decreased CXCL12 (SDF-1) plasma levels in early Alzheimer's disease: a contribution to a deficient hematopoietic brain support? J Alzheimers Dis 2008;15:83-95.

**23**. O'Bryant SE, Hobson V, Hall JR, et al. Brain-derived neurotrophic factor levels in Alzheimer's disease. J Alzheimers Dis 2009;17:337-341.

**24**. Reale M, Kamal MA, Velluto L, Gambi D, Di Nicola M, Greig NH. Relationship between inflammatory mediators, Abeta levels and ApoE genotype in Alzheimer disease. Curr Alzheimer Res 2012;9:447-457.

**25**. Baranowska-Bik A, Bik W, Wolinska-Witort E, et al. Plasma beta amyloid and cytokine profile in women with Alzheimer's disease. Neuro Endocrinol Lett 2008;29:75-79.

**26**. Alvarez XA, Franco A, Fernandez-Novoa L, Cacabelos R. Blood levels of histamine, IL-1 beta, and TNF-alpha in patients with mild to moderate Alzheimer disease. Mol Chem Neuropathol 1996;29:237-252.

**27**. Blum-Degen D, Muller T, Kuhn W, Gerlach M, Przuntek H, Riederer P. Interleukin-1 beta and interleukin-6 are elevated in the cerebrospinal fluid of Alzheimer's and de novo Parkinson's disease patients. Neurosci Lett 1995;202:17-20.

**28**. Bruunsgaard H, Andersen-Ranberg K, Jeune B, Pedersen AN, Skinhoj P, Pedersen BK. A high plasma concentration of TNF-alpha is associated with dementia in centenarians. J Gerontol A Biol Sci Med Sci 1999;54:M357-364.

**29**. Tarkowski E, Blennow K, Wallin A, Tarkowski A. Intracerebral production of tumor necrosis factor-alpha, a local neuroprotective agent, in Alzheimer disease and vascular dementia. J Clin Immunol 1999;19:223-230.

**30**. Maes M, DeVos N, Wauters A, et al. Inflammatory markers in younger vs elderly normal volunteers and in patients with Alzheimer's disease. J Psychiatr Res 1999;33:397-405.

**31**. Angelis P, Scharf S, Mander A, Vajda F, Christophidis N. Serum interleukin-6 and interleukin-6 soluble receptor in Alzheimer's disease. Neurosci Lett 1998;244:106-108.

**32**. Chao CC, Ala TA, Hu S, et al. Serum cytokine levels in patients with Alzheimer's disease. Clin Diagn Lab Immunol 1994;1:433-436.

**33**. van Duijn CM, Hofman A, Nagelkerken L. Serum levels of interleukin-6 are not elevated in patients with Alzheimer's disease. Neurosci Lett 1990;108:350-354.

**34**. Chen R, Yin Y, Zhao Z, et al. Elevation of serum TNF-alpha levels in mild and moderate Alzheimer patients with daytime sleepiness. J Neuroimmunol 2012;244:97-102.

**35**. Juraskova B, Andrys C, Holmerova I, et al. Transforming growth factor beta and soluble endoglin in the healthy senior and in Alzheimer's disease patients. J Nutr Health Aging 2010;14:758-761.

**36**. Kester MI, van der Flier WM, Visser A, Blankenstein MA, Scheltens P, Oudejans CB. Decreased mRNA expression of CCL5 [RANTES] in Alzheimer's disease blood samples. Clin Chem Lab Med 2012;50:61-65.

**37**. Forlenza OV, Diniz BS, Talib LL, et al. Increased serum IL-1beta level in Alzheimer's disease and mild cognitive impairment. Dement Geriatr Cogn Disord 2009;28:507-512.

**38**. Marksteiner J, Kemmler G, Weiss EM, et al. Five out of 16 plasma signaling proteins are enhanced in plasma of patients with mild cognitive impairment and Alzheimer's disease. Neurobiol Aging 2011;32:539-540.

**39**. Hernanz A, De la Fuente M, Navarro M, Frank A. Plasma aminothiol compounds, but not serum tumor necrosis factor receptor II and soluble receptor for advanced glycation end products, are related to the cognitive impairment in Alzheimer's disease and mild cognitive impairment patients. Neuroimmunomodulation 2007;14:163-167.

**40**. Rota E, Bellone G, Rocca P, Bergamasco B, Emanuelli G, Ferrero P. Increased intrathecal TGF-beta1, but not IL-12, IFN-gamma and IL-10 levels in Alzheimer's disease patients. Neurol Sci 2006;27:33-39.

**41**. Alvarez A, Cacabelos R, Sanpedro C, Garcia-Fantini M, Aleixandre M. Serum TNF-alpha levels are increased and correlate negatively with free IGF-I in Alzheimer disease. Neurobiol Aging 2007;28:533-536.

**42**. Galimberti D, Fenoglio C, Lovati C, et al. Serum MCP-1 levels are increased in mild cognitive impairment and mild Alzheimer's disease. Neurobiol Aging 2006;27:1763-1768.

**43**. Lindberg C, Chromek M, Ahrengart L, Brauner A, Schultzberg M, Garlind A. Soluble interleukin-1 receptor type II, IL-18 and caspase-1 in mild cognitive impairment and severe Alzheimer's disease. Neurochem Int 2005;46:551-557.

**44**. Solerte SB, Cravello L, Ferrari E, Fioravanti M. Overproduction of IFN-gamma and TNF-alpha from natural killer (NK) cells is associated with abnormal NK reactivity and cognitive derangement in Alzheimer's disease. Ann N Y Acad Sci 2000;917:331-340.

**45**. O'Bryant SE, Waring SC, Hobson V, et al. Decreased C-reactive protein levels in Alzheimer disease. J Geriatr Psychiatry Neurol 2010;23:49-53.

**46**. Hasegawa Y, Sawada M, Ozaki N, Inagaki T, Suzumura A. Increased soluble tumor necrosis factor receptor levels in the serum of elderly people. Gerontology 2000;46:185-188.

**47**. Tarkowski E, Andreasen N, Tarkowski A, Blennow K. Intrathecal inflammation precedes development of Alzheimer's disease. J Neurol Neurosurg Psychiatry 2003;74:1200-1205.

**48**. Lanzrein AS, Johnston CM, Perry VH, Jobst KA, King EM, Smith AD. Longitudinal study of inflammatory factors in serum, cerebrospinal fluid, and brain tissue in Alzheimer disease: interleukin-1beta, interleukin-6, interleukin-1 receptor antagonist, tumor necrosis factor-alpha, the soluble tumor necrosis factor receptors I and II, and alpha1-antichymotrypsin. Alzheimer Dis Assoc Disord 1998;12:215-227.

**49**. Lepara O, Alajbegovic A, Zaciragic A, et al. Elevated serum homocysteine level is not associated with serum C-reactive protein in patients with probable Alzheimer's disease. J Neural Transm 2009;116:1651-1656.

**50**. Mashayekhi F, Hadavi M, Vaziri HR, Naji M. Increased acidic fibroblast growth factor concentrations in the serum and cerebrospinal fluid of patients with Alzheimer's disease. J Clin Neurosci 2010;17:357-359.

**51**. Engelborghs S, De Brabander M, De Cree J, et al. Unchanged levels of interleukins, neopterin, interferon-gamma and tumor necrosis factor-alpha in cerebrospinal fluid of patients with dementia of the Alzheimer type. Neurochem Int 1999;34:523-530.

**52**. Fillit H, Ding WH, Buee L, et al. Elevated circulating tumor necrosis factor levels in Alzheimer's disease. Neurosci Lett 1991;129:318-320.

**53**. Garlind A, Brauner A, Hojeberg B, Basun H, Schultzberg M. Soluble interleukin-1 receptor type II levels are elevated in cerebrospinal fluid in Alzheimer's disease patients. Brain Res 1999;826:112-116.

**54**. Craig-Schapiro R, Kuhn M, Xiong C, et al. Multiplexed immunoassay panel identifies novel CSF biomarkers for Alzheimer's disease diagnosis and prognosis. PLoS One 2011;6:e18850.

**55**. Hampel H, Schoen D, Schwarz MJ, et al. Interleukin-6 is not altered in cerebrospinal fluid of first-degree relatives and patients with Alzheimer's disease. Neurosci Lett 1997;228:143-146.

**56**. Hampel H, Teipel SJ, Padberg F, et al. Discriminant power of combined cerebrospinal fluid tau protein and of the soluble interleukin-6 receptor complex in the diagnosis of Alzheimer's disease. Brain Res 1999;823:104-112.

**57**. Harigaya Y, Shoji M, Nakamura T, Matsubara E, Hosoda K, Hirai S. Alpha 1-antichymotrypsin level in cerebrospinal fluid is closely associated with late onset Alzheimer's disease. Intern Med 1995;34:481-484.

**58**. Licastro F, Morini MC, Polazzi E, Davis LJ. Increased serum alpha 1-antichymotrypsin in patients with probable Alzheimer's disease: an acute phase reactant without the peripheral acute phase response. J Neuroimmunol 1995;57:71-75.

**59**. Licastro F, Masliah E, Pedrini S, Thal LJ. Blood levels of alpha-1-antichymotrypsin and risk factors for Alzheimer's disease: effects of gender and apolipoprotein E genotype. Dement Geriatr Cogn Disord 2000;11:25-28.

**60**. Martinez M, Frank A, Hernanz A. Relationship of interleukin-1 beta and beta 2-microglobulin with neuropeptides in cerebrospinal fluid of patients with dementia of the Alzheimer type. J Neuroimmunol 1993;48:235-240.

**61**. Martinez M, Fernandez-Vivancos E, Frank A, De la Fuente M, Hernanz A. Increased cerebrospinal fluid fas (Apo-1) levels in Alzheimer's disease. Relationship with IL-6 concentrations. Brain Res 2000;869:216-219.

**62**. Marz P, Heese K, Hock C, et al. Interleukin-6 (IL-6) and soluble forms of IL-6 receptors are not altered in cerebrospinal fluid of Alzheimer's disease patients. Neurosci Lett 1997;239:29-32.

**63**. Doecke JD, Laws SM, Faux NG, et al. Blood-Based Protein Biomarkers for Diagnosis of Alzheimer Disease. Arch Neurol 2012:1-8.

**64**. Blasko I, Lederer W, Oberbauer H, et al. Measurement of thirteen biological markers in CSF of patients with Alzheimer's disease and other dementias. Dement Geriatr Cogn Disord 2006;21:9-15.

**65**. Pirttila T, Mehta PD, Frey H, Wisniewski HM. Alpha 1-antichymotrypsin and IL-1 beta are not increased in CSF or serum in Alzheimer's disease. Neurobiol Aging 1994;15:313-317.

**66**. Yamada K, Kono K, Umegaki H, et al. Decreased interleukin-6 level in the cerebrospinal fluid of patients with Alzheimer-type dementia. Neurosci Lett 1995;186:219-221.

**67**. Licastro F, Parnetti L, Morini MC, et al. Acute phase reactant alpha 1-antichymotrypsin is increased in cerebrospinal fluid and serum of patients with probable Alzheimer disease. Alzheimer Dis Assoc Disord 1995;9:112-118.

**68**. Hampel H, Sunderland T, Kotter HU, et al. Decreased soluble interleukin-6 receptor in cerebrospinal fluid of patients with Alzheimer's disease. Brain Res 1998;780:356-359.

**69**. Angelucci F, Spalletta G, di Iulio F, et al. Alzheimer's disease (AD) and Mild Cognitive Impairment (MCI) patients are characterized by increased BDNF serum levels. Curr Alzheimer Res 2010;7:15-20.

**70**. Li G, Peskind ER, Millard SP, et al. Cerebrospinal fluid concentration of brain-derived neurotrophic factor and cognitive function in non-demented subjects. PLoS One 2009;4:e5424.

**71**. Angelopoulos P, Agouridaki H, Vaiopoulos H, et al. Cytokines in Alzheimer's disease and vascular dementia. Int J Neurosci 2008;118:1659-1672.

**72**. Buchhave P, Janciauskiene S, Zetterberg H, Blennow K, Minthon L, Hansson O. Elevated plasma levels of soluble CD40 in incipient Alzheimer's disease. Neurosci Lett 2009;450:56-59.

**73**. Murase K, Nabeshima T, Robitaille Y, Quirion R, Ogawa M, Hayashi K. NGF level of is not decreased in the serum, brain-spinal fluid, hippocampus, or parietal cortex of individuals with Alzheimer's disease. Biochem Biophys Res Commun 1993;193:198-203.

**74**. Lieberman J, Schleissner L, Tachiki KH, Kling AS. Serum alpha 1-antichymotrypsin level as a marker for Alzheimer-type dementia. Neurobiol Aging 1995;16:747-753.

**75**. DeKosky ST, Ikonomovic MD, Wang X, et al. Plasma and cerebrospinal fluid alpha1-antichymotrypsin levels in Alzheimer's disease: correlation with cognitive impairment. Ann Neurol 2003;53:81-90.

**76**. Han Y, Jia J, Jia XF, Qin W, Wang S. Combination of plasma biomarkers and clinical data for the detection of sporadic Alzheimer's disease. Neurosci Lett 2012;516:232-236.

**77**. Licastro F, Pedrini S, Davis LJ, et al. Alpha-1-antichymotrypsin and oxidative stress in the peripheral blood from patients with probable Alzheimer disease: a short-term longitudinal study. Alzheimer Dis Assoc Disord 2001;15:51-55.

**78**. O'Bryant SE, Hobson VL, Hall JR, et al. Serum brain-derived neurotrophic factor levels are specifically associated with memory performance among Alzheimer's disease cases. Dement Geriatr Cogn Disord 2011;31:31-36.

**79**. De Servi B, La Porta CA, Bontempelli M, Comolli R. Decrease of TGF-beta1 plasma levels and increase of nitric oxide synthase activity in leukocytes as potential biomarkers of Alzheimer's disease. Exp Gerontol 2002;37:813-821.

**80**. Dicou E, Vermersch P, Penisson-Besnier I, Dubas F, Nerriere V. Anti-NGF autoantibodies and NGF in sera of Alzheimer patients and in normal subjects in relation to age. Autoimmunity 1997;26:189-194.

**81**. Laske C, Stransky E, Leyhe T, et al. Stage-dependent BDNF serum concentrations in Alzheimer's disease. J Neural Transm 2006;113:1217-1224.

**82**. Laske C, Stransky E, Leyhe T, et al. BDNF serum and CSF concentrations in Alzheimer's disease, normal pressure hydrocephalus and healthy controls. J Psychiatr Res 2007;41:387-394.

**83**. Bonotis K, Krikki E, Holeva V, Aggouridaki C, Costa V, Baloyannis S. Systemic immune aberrations in Alzheimer's disease patients. J Neuroimmunol 2008;193:183-187.

**84**. Teunissen CE, Lutjohann D, von Bergmann K, et al. Combination of serum markers related to several mechanisms in Alzheimer's disease. Neurobiol Aging 2003;24:893-902.

**85**. Laske C, Stellos K, Hoffmann N, et al. Higher BDNF serum levels predict slower cognitive decline in Alzheimer's disease patients. Int J Neuropsychopharmacol 2011;14:399-404.

**86**. Inspector M, Aharon-Perez J, Glass-Marmor L, Miller A. Matrix metalloproteinase-9, its tissue inhibitor(TIMP)-1 and CRP in Alzheimer's disease. Eur Neurol 2005;53:155-157.

**87**. Zuliani G, Cavalieri M, Galvani M, et al. Markers of endothelial dysfunction in older subjects with late onset Alzheimer's disease or vascular dementia. J Neurol Sci 2008;272:164-170.

**88**. Genc S, Egrilmez MY, Yaka E, et al. TNF-related apoptosis-inducing ligand level in Alzheimer's disease. Neurol Sci 2009;30:263-267.

**89**. Laske C, Stransky E, Hoffmann N, et al. Macrophage colony-stimulating factor (M-CSF) in plasma and CSF of patients with mild cognitive impairment and Alzheimer's disease. Curr Alzheimer Res 2010;7:409-414.

**90**. Yasutake C, Kuroda K, Yanagawa T, Okamura T, Yoneda H. Serum BDNF, TNF-alpha and IL-1beta levels in dementia patients: comparison between Alzheimer's disease and vascular dementia. Eur Arch Psychiatry Clin Neurosci 2006;256:402-406.

**91**. Choi C, Jeong JH, Jang JS, et al. Multiplex analysis of cytokines in the serum and cerebrospinal fluid of patients with Alzheimer's disease by color-coded bead technology. J Clin Neurol 2008;4:84-88.

**92**. Lawlor BA, Swanwick GR, Feighery C, Walsh JB, Coakley D. Acute phase reactants in Alzheimer's disease. Biol Psychiatry 1996;39:1051-1052.

**93**. Mulder SD, Heijst JA, Mulder C, et al. CSF levels of PSA and PSA-ACT complexes in Alzheimer's disease. Ann Clin Biochem 2009;46:477-483.

**94**. Chao CC, Hu S, Frey WH, 2nd, Ala TA, Tourtellotte WW, Peterson PK. Transforming growth factor beta in Alzheimer's disease. Clin Diagn Lab Immunol 1994;1:109-110.

**95**. Laske C, Stellos K, Stransky E, Leyhe T, Gawaz M. Decreased plasma levels of granulocyte-colony stimulating factor (G-CSF) in patients with early Alzheimer's disease. J Alzheimers Dis 2009;17:115-123.

**96**. Helmy AA, Naseer MM, Shafie SE, Nada MA. Role of interleukin 6 and alpha-globulins in differentiating Alzheimer and vascular dementias. Neurodegener Dis 2012;9:81-86.

**97**. Cojocaru IM, Cojocaru M, Miu G, Sapira V. Study of interleukin-6 production in Alzheimer's disease. Rom J Intern Med 2011;49:55-58.

**98**. Jia JP, Meng R, Sun YX, Sun WJ, Ji XM, Jia LF. Cerebrospinal fluid tau, Abeta1-42 and inflammatory cytokines in patients with Alzheimer's disease and vascular dementia. Neurosci Lett 2005;383:12-16.

**99**. Rosler N, Wichart I, Jellinger KA. Clinical significance of neurobiochemical profiles in the lumbar cerebrospinal fluid of Alzheimer's disease patients. J Neural Transm 2001;108:231-246.

**100**. Gomez-Tortosa E, Gonzalo I, Fanjul S, et al. Cerebrospinal fluid markers in dementia with lewy bodies compared with Alzheimer disease. Arch Neurol 2003;60:1218-1222.

**101**. Galimberti D, Venturelli E, Fenoglio C, et al. Intrathecal levels of IL-6, IL-11 and LIF in Alzheimer's disease and frontotemporal lobar degeneration. J Neurol 2008;255:539-544.

**102**. Tarkowski E, Issa R, Sjogren M, et al. Increased intrathecal levels of the angiogenic factors VEGF and TGF-beta in Alzheimer's disease and vascular dementia. Neurobiol Aging 2002;23:237-243.

**103**. Zetterberg H, Andreasen N, Blennow K. Increased cerebrospinal fluid levels of transforming growth factor-beta1 in Alzheimer's disease. Neurosci Lett 2004;367:194-196.

**104**. Correa JD, Starling D, Teixeira AL, Caramelli P, Silva TA. Chemokines in CSF of Alzheimer's disease patients. Arq Neuropsiquiatr 2011;69:455-459.

**105**. Ozturk C, Ozge A, Yalin OO, et al. The diagnostic role of serum inflammatory and soluble proteins on dementia subtypes: correlation with cognitive and functional decline. Behav Neurol 2007;18:207-215.

**106**. Malaguarnera L, Motta M, Di Rosa M, Anzaldi M, Malaguarnera M. Interleukin-18 and transforming growth factor-beta 1 plasma levels in Alzheimer's disease and vascular dementia. Neuropathology 2006;26:307-312.

**107**. Matsubara E, Amari M, Shoji M, et al. Serum concentration of alpha 1-antichymotrypsin is elevated in patients with senile dementia of the Alzheimer type. Prog Clin Biol Res 1989;317:707-714.

**108**. Cacabelos R, Alvarez XA, Franco-Maside A, Fernandez-Novoa L, Caamano J. Serum tumor necrosis factor (TNF) in Alzheimer's disease and multi-infarct dementia. Methods Find Exp Clin Pharmacol 1994;16:29-35.

**109**. Rodriguez-Rodriguez E, Sanchez-Juan P, Mateo I, et al. Serum levels and genetic variation of TGF-beta1 are not associated with Alzheimer's disease. Acta Neurol Scand 2007;116:409-412.

**110**. De Luigi A, Pizzimenti S, Quadri P, et al. Peripheral inflammatory response in Alzheimer's disease and multiinfarct dementia. Neurobiol Dis 2002;11:308-314.

**111**. Diniz BS, Teixeira AL, Ojopi EB, et al. Higher serum sTNFR1 level predicts conversion from mild cognitive impairment to Alzheimer's disease. J Alzheimers Dis 2010;22:1305-1311.

**112**. Bonaccorso S, Lin A, Song C, et al. Serotonin-immune interactions in elderly volunteers and in patients with Alzheimer's disease (DAT): lower plasma tryptophan availability to the brain in the elderly and increased serum interleukin-6 in DAT. Aging (Milano) 1998;10:316-323.

**113**. Kalman J, Juhasz A, Laird G, et al. Serum interleukin-6 levels correlate with the severity of dementia in Down syndrome and in Alzheimer's disease. Acta Neurol Scand 1997;96:236-240.

**114**. Vom Berg J, Prokop S, Miller KR, et al. Inhibition of IL-12/IL-23 signaling reduces Alzheimer's disease-like pathology and cognitive decline. Nat Med 2012;18:1812-1819.

**115**. Zhang R, Miller RG, Madison C, et al. Systemic immune system alterations in early stages of Alzheimer's disease. J Neuroimmunol 2013;256:38-42.

**116**. Huang L, Jia J, Liu R. Decreased serum levels of the angiogenic factors VEGF and TGF-beta1 in Alzheimer's disease and amnestic mild cognitive impairment. Neurosci Lett 2013;550:60-63.

**117**. Soares HD, Chen Y, Sabbagh M, Roher A, Schrijvers E, Breteler M. Identifying early markers of Alzheimer's disease using quantitative multiplex proteomic immunoassay panels. Ann N Y Acad Sci 2009;1180:56-67.

**118**. Doecke JD, Laws SM, Faux NG, et al. Blood-based protein biomarkers for diagnosis of Alzheimer disease. Arch Neurol 2012;69:1318-1325.
