# Supplementary material for: Body Fluid Cytokine Levels in Mild Cognitive Impairment and Alzheimer’s Disease: a Comparative Overview
Source: Mol Neurobiol. 2014 Feb 25;50(2):534–44. doi: 10.1007/s12035-014-8657-1 (PMC4182618; doi:10.1007/s12035-014-8657-1)
Supplement: Supplementary file 2 — (DOCX 72 kb) [file 12035_2014_8657_MOESM2_ESM.docx]

**Supplementary 2: Overview of regulation of cytokines and other inflammation associated proteins in MCI and AD**

This table gives an overview of the regulation of cytokines, chemokines, their receptors and other proteins associated with immune signaling and immune reactions as observed in plasma/serum and CSF of AD and MCI patients. It contains 66 proteins described in the 118 articles included in the supplementary reference list (see below) along with their most common synonyms, direction of regulation and the method used for quantification. **↑**= up regulated, **↓** = down regulated, ▬ = no regulation, P.d.R. = disease progression dependent regulation (correlation of protein levels with MCI to AD turnover or with cognitive decline). For brief descriptions of the respective articles, see supplementary 1.

| **Protein** | **Synonyms** | **Alzheimer´s disease (AD)** | | **Mild cognitive impairment (MCI)** | | **Method** |
| --- | --- | --- | --- | --- | --- | --- |
|  |  | **Plasma/Serum** | **CSF** | **Plasma/Serum** | **CSF** |  |
| **ACT** | Alpha1-Antichymotrypsin, Serpin A3 | **↑**(8, 58, 59, 74, 75, 76, 77, 107, 114)  ▬(48, 65, 67, 92, 93, 105) | **↑** (57, 67, 75)  ▬ (48, 65, 93) | ▬ (105) |  | ELISA (48, 57, 58, 65, 67, 76, 93, 105)  Immunodiffusion (8, 59, 74, 75, 77, 92, 107) |
| **ANG-2** | Angiopoietin-2 | **↑** (1)  ▬ (6, 38) |  | ▬ (38) |  | Cytokine Array (1, 6, 38) |
| **BDNF** | Brain-derived neurotrophic factor | **↑** (69)  **↓**(82, 90)  ▬ (23, 78)  P.d.R. (81, 85) | **↓** (70)  ▬ (64, 82) | **↑** (69) | ▬ (70) | ELISA (23, 64, 69, 70, 81, 82, 85, 90)  Multiplex Assay (78) |
| **β-NGF** | Beta-nerve growth factor, NGF, NGFB | ▬ (17, 73, 80) | ▬ (73) | ▬ (17) |  | ELISA (17, 73, 80) |
| **CCL2 / MCP-1** | Monocyte chemoattractant protein 1 | **↑** (15)  **↓** (24)  ▬ (11, 16, 17, 91) | **↑** (16, 18, 104)  ▬ (91)  P.d.R. (42) | ▬ (2, 11) | **↑** (18)  ▬ (2)  P.d.R. (42) | Cytokine Array (15)  ELISA (2, 11, 16, 17. 18, 24, 42, 104)  Multiplex Assay (91) |
| **CCL3 / MIP-1α** | Macrophage inflammatory protein 1-alpha, SCYA3 | **↑** (63, 118)  ▬ (91) | ▬ (64) |  |  | ELISA (64, 91)  Multiplex Assay (63, 118) |
| **Protein** | **Synonyms** | **Alzheimer´s disease (AD)** | | **Mild cognitive impairment (MCI)** | | **Method** |
|  |  | **Plasma/Serum** | **CSF** | **Plasma/Serum** | **CSF** |  |
| **CCL5 / RANTES** | SCYA5 | **↑** (24, 38, 117)  **↓** (1, 36)  ▬ (9, 91) |  | **↑** (38) |  | Cytokine Array (1, 9, 38)  ELISA (24, 91)  qRT-PCR (36)  Multiplex Assay (117) |
| **CCL7 / MCP-3** | Monocyte chemoattractant protein, Monocyte chemotactic protein 3, SCYA6, SCYA7 | **↓** (1)  ▬ (6, 17, 38) |  | ▬ (17, 38) |  | Cytokine Array (1, 6, 17, 38) |
| **CCL11 / Eotaxin** | SCYA11 | **↓** (91) |  | ▬ (2) | ▬ (2) | ELISA (2)  Multiplex Assay (91) |
| **CCL13 / MCP-4** | Monocyte chemoattractant protein 4, SCYA13 |  |  | ▬ (2) | ▬ (2) | ELISA (2) |
| **CCL15 / MIP-1δ** | Macrophage-inflammatory protein 1 delta, Macrophage inflammatory protein 5 (MIP-5), SCYA15 | **↑** (6, 38)  **↓** (1) |  | ▬ (38) |  | Cytokine Array (1, 6, 38) |
| **CCL18 / MIP-4** | Macrophage inflammatory protein 4, SCYA18 | ▬ (1, 6, 38) |  | **↑** (38) |  | Cytokine Array (1, 6, 38) |
| **CCL26 / MIP-4α** | Eotaxin-3, Macrophage inflammatory protein 4-alpha, SCYA26 |  |  | ▬ (2) | **↑** (54)  P.d.R. (2) | ELISA (2)  Multiplex Assay (54) |
| **CRP** | C-reactive protein, PTX1 | **↑** (96)  **↓** (45)  ▬ (8, 49, 59, 77, 84, 86, 92) |  |  |  | ELISA (84, 86, 96)  Immunodiffusion (8, 59, 77, 92)  Immunoephelometrie (49)  Multiplex Assay (45) |
| **CTACK** | Cutaneous T-cell-attracting chemokine, SCYA27 | **↑** (17) |  | ▬ (17) |  | ELISA (17) |
| **EGF** | Pro-epidermal growth factor | **↑** (6, 38)  **↓** (1)  ▬ (15) |  | ▬ (38) |  | Cytokine Array (1, 6, 15, 38) |
| **Protein** | **Synonyms** | **Alzheimer´s disease (AD)** | | **Mild cognitive impairment (MCI)** | | **Method** |
|  |  | **Plasma/Serum** | **CSF** | **Plasma/Serum** | **CSF** |  |
| **E-Selectin** | Endothelial leukocyte adhesion molecule 1, ELAM-1 | ▬ (15, 87) |  |  |  | Cytokine Array (15)  ELISA (87) |
| **FGF1** | Fibroblast growth factor 1, Acidic fibroblast growth factor, aFGF | **↑** (50) | **↑** (50) |  |  | ELISA (50) |
| **FGF2** | Fibroblast growth factor 2, Basic fibroblast growth factor, bFGF |  | ▬ (64) |  |  | ELISA (64) |
| **G-CSF** | Granulocyte colony-stimulating factor | **↓** (1, 95)  ▬ (6, 46, 38) |  | ▬ (38) |  | Cytokine Array (1, 6, 38)  ELISA (46, 95) |
| **GDNF** | Glial cell line-derived neurotrophic factor, Astrocyte-derived trophic factor, ATF | **↑** (38)  **↓** (1)  ▬ (6) | ▬ (64) | ▬ (38) |  | Cytokine Array (1, 6, 38)  ELISA (64) |
| **GM-CSF** | Granulocyte-macrophage colony-stimulating factor | ▬ (4, 91) | ▬ (5) |  |  | ELISA (4, 5)  Multiplex Assay (91) |
| **GRO-a** | Growth-regulated alpha protein, CXCL1 | ▬ (17) |  | ▬ (17) |  | ELISA (17) |
| **HGF** | Hepatocyte growth factor | ▬ (17) | ▬ (64) | ▬ (17) |  | ELISA (17, 64) |
| **ICAM-1** | Intercellular adhesion molecule 1, CD54 | **↑** (1, 24)  ▬ (6, 17, 36, 38)  **↓** (117) |  | **↑** (38)  ▬ (17) |  | Cytokine Array (1, 6, 38)  ELISA (17, 24)  qRt-PCR (36)  Multiplex-Assay (117) |
| **IFN-α** | Interferon alpha | **↑** (12)  ▬ (17, 19) |  | **↑** (12)  ▬ (17) |  | ELISA (12, 17, 19) |
| **Protein** | **Synonyms** | **Alzheimer´s disease (AD)** | | **Mild cognitive impairment (MCI)** | | **Method** |
|  |  | **Plasma/Serum** | **CSF** | **Plasma/Serum** | **CSF** |  |
| **IFN-γ** | Interferon gamma | **↑** (15)  ▬ (4, 19, 44, 87, 91) | ▬ (4, 51) |  |  | Cytokine Array (15)  ELISA (4, 19, 44, 51, 87)  Multiplex Assay (91) |
| **IGF-I** | Insulin-like growth factor I | **↓** (41) |  | **↓** (41) |  | ELISA (41) |
| **IL-1α** | Interleukin-1 alpha | **↓** (1)  ▬ (6, 15, 17, 36, 38, 83, 91), |  | ▬ (17, 38) |  | Cytokine Array (1, 6, 15, 38)  ELISA (17, 83)  Multiplex Assay (91)  qRT-PCR (36) |
| **IL-1β** | Interleukin-1 beta | **↑** (8, 14, 26, 37, 71, 87, 110)  ▬ (4, 10, 15, 29, 48, 65, 90, 105) | **↑** (27)  ▬ (4, 20, 29, 47, 48, 51, 60, 61, 65, 100) | **↑** (37) | ▬ (47) | Cytokine Array (15)  ELISA (4, 8, 10, 14, 20, 27, 29, 37, 47, 48, 51, 61, 65, 71, 87, 90, 100, 105, 110)  Radioimmunoassay (26, 60) |
| **IL1RA** | Interleukin-1 receptor antagonist protein | ▬ (10, 48) | ▬ (48) |  |  | ELISA (10, 48) |
| **IL-1RII** | Interleukin-1 receptor type 2, CD121b | ▬ (43) | **↑** (53)  ▬ (43) | ▬ (43) | ▬ (43) | ELISA (43, 53) |
| **IL-2** | Interleukin-2 | ▬ (4, 15, 27, 71, 83, 105, 110) | ▬ (4, 27, 98) |  |  | Cytokine Array (15)  ELISA (4, 27, 71, 83, 98, 105, 110) |
| **IL-2R** | Interleukin-2 receptor, heterotrimer of IL-2R alpha, IL2-R beta and IL-2R gamma. | ▬ (17, 20) | ▬ (20, 51) | ▬ (17) |  | ELISA (17, 20, 51) |
| **IL-3** | Interleukin-3 | **↑** (117)  **↓** (1)  ▬ (6, 17, 36, 38) |  | ▬ (17, 38) |  | Cytokine Array (1, 6, 38)  ELISA (17)  qRT-PCR (36)  Multiplex-Assay (117) |
| **Protein** | **Synonyms** | **Alzheimer´s disease (AD)** | | **Mild cognitive impairment (MCI)** | | **Method** |
|  |  | **Plasma/Serum** | **CSF** | **Plasma/Serum** | **CSF** |  |
| **IL-6** | Interleukin-6 | **↑** (8, 12, 19, 25, 27, 29, 30, 71, 96, 97, 112)  **↓** (20)  ▬ (4, 14, 15, 31, 32, 33, 73, 48, 83, 84, 87, 105)  P.d.R. (113) | **↑** (27, 61, 98, 99)  **↓** (66)  ▬ (4, 20, 29, 73, 48, 51, 53, 55, 56, 62, 100, 101, 113) | ▬(12) |  | Cytokine Array (15)  Cell-based bioassay (32, 33)  ELISA (4, 8, 12, 14, 19, 20, 25, 27, 29, 30, 31, 48, 51, 53, 55, 56, 61, 62, 66, 71, 73, 83, 84, 87, 96, 97, 98, 99, 100, 101, 105, 112, 113) |
| **IL-6R** | Interleukin-6 receptor subunit alpha, CD126 | **↓** (31, 84)  ▬ (20, 46) | **↓** (56, 68)  ▬ (20, 62) |  |  | ELISA (20, 31, 46, 56, 62, 68, 84) |
| **IL-7** | Interleukin-7 | ▬ (91) |  |  | **↓** (54) | Multiplex Assay (54, 91) |
| **IL-8 / CXCL8** | Interleukin-8 | **↑** (15, 117)  **↓** (11)  ▬ (1, 4, 6, 9, 16, 36, 38, 83, 91, 110, 112) | **↑** (16, 18)  ▬ (4, 104) | **↓** (11)  ▬ (9, 38) | **↑** (18) | Cytokine Array (1, 6, 15, 38)  ELISA (4, 9, 11, 16, 18, 83, 91, 104, 110, 112)  qRT-PCR (36)  Multiplex Assay (117) |
| **IL-10** | Interleukin-10 | **↑** (71, 118)  ▬ (4, 10, 11, 14, 25, 40, 83) | ▬ (4, 40, 51) | ▬ (11, 83) | **↑** (54) | ELISA (4, 10,11, 14, 25, 40, 51, 71, 83)  Multiplex Assay (54, 118) |
| **IL-11** | Interleukin-11 | **↑** (1)  ▬ (6, 38) | **↑** (101) | ▬ (38) |  | Cytokine Array (1, 6, 38)  ELISA (101) |
| **IL-12** | Interleukin-12, heterodimer of IL-12A und IL-12B | ▬ (4, 17, 19, 40, 91)  P.d.R.(13) | ▬ (4, 40, 51)  P.d.R. (114) | ▬ (17) |  | ELISA (4,13, 17, 19, 40, 51, 114)  Multiplex Assay (91) |
| **IL-16** | Pro-interleukin-16 | ▬ (17)  P.d.R.(13) |  | ▬ (17) |  | ELISA (13, 17) |
| **IL-18** | Interleukin-18 | **↑** (24, 106)  ▬ (7, 17, 43, 105)  P.d.R.(13) | **↑** (7) | ▬ (17, 43, 105) |  | ELISA (7, 13, 17, 24, 43, 105, 106)  WB (7) |
| **Protein** | **Synonyms** | **Alzheimer´s disease (AD)** | | **Mild cognitive impairment (MCI)** | | **Method** |
|  |  | **Plasma/Serum** | **CSF** | **Plasma/Serum** | **CSF** |  |
| **IL-18BP** | Interleukin-18-binding protein | **↓** (24) |  |  |  | ELISA (24) |
| **IP-10 / CXCL10** | 10 kDa interferon gamma-induced protein | ▬ (16, 17, 91) | **↑** (16, 18)  ▬ (104) | ▬ (17) | **↑** (18, 54) | ELISA (16, 17, 18, 91, 104)  Multiplex Assay (54) |
| **LIF** | Leukemia inhibitory factor | ▬ (17) |  | ▬ (17) |  | ELISA (17) |
| **L-selectin** | Leukocyte adhesion molecule 1 | **↓** (15) |  |  |  | Cytokine Array (15) |
| **M-CSF** | Macrophage colony-stimulating factor 1 | **↑** (46, 89)  **↓** (1)  ▬ (6, 17, 38) | ▬ (89) | ▬ (17, 38) | **↓** (89) | Cytokine Array (1, 6, 38)  ELISA (17, 46, 89) |
| **MIF** | Macrophage migration inhibitory factor | **↑** (17) |  | **↑** (17) | **↑** (54) | ELISA (17)  Multiplex Assay (54) |
| **MIG / CXCL9** | Monokine induced by interferon-gamma | **↑** (17) |  | ▬ (17) | **↑** (54) | ELISA (17)  Multiplex Assay (54) |
| **PDGF-BB** | Platelet-derived growth factor subunit B, homodimer | **↑** (6)  **↓** (1)  ▬ (36, 38) |  | ▬ (38) |  | Cytokine Array (1, 6, 38)  qRT-PCR (36) |
| **P-selectin** | Leukocyte-endothelial cell adhesion molecule 3, CD62P | **↓** (15) |  |  |  | Cytokine Array (15) |
| **soluble CD40** | Tumor necrosis factor receptor superfamily member 5, TNFRSF5, CD40 ligand receptor, CD40 | **↑** (3, 5, 63, 118) |  | P.d.R. (72) |  | ELISA (5, 72)  Multiplex Assay (63, 118)  WB (3) |
| **Protein** | **Synonyms** | **Alzheimer´s disease (AD)** | | **Mild cognitive impairment (MCI)** | | **Method** |
|  |  | **Plasma/Serum** | **CSF** | **Plasma/Serum** | **CSF** |  |
| **CD40L** | CD40 ligand, CD154 | **↑** (5) |  | ▬ (72) |  | ELISA (5, 72) |
| **SCF** | Stem cell factor | **↑** (17) |  | ▬ (17) |  | ELISA (17) |
| **SCGF** | Stem cell growth factor | ▬ (17) |  | ▬ (17) |  | ELISA (17) |
| **SDF-1a** | Stromal cell-derived factor 1 | ↓(22)  ▬ (17) | ▬ (22) | ▬ (17) |  | ELISA (17, 22) |
| **TNF-α** | Tumor necrosis factor alpha | **↑** (10, 12, 14, 21, 28, 29, 34, 38, 41, 52, 83, 87, 117)  **↓** (1, 11, 26, 30, 108)  ▬ (4, 6, 15, 25, 32, 34, 36, 44, 48, 71, 90, 91, 105, 110, 111) | **↑** (29, 47, 98)  **↓** (20)  ▬ (4, 48, 51, 53, 64) | **↑** (38, 41)  ▬ (11,12, 111) | **↓** (47) | Cell-based bioassay (32, 52)  Cytokine Assay (1, 11, 15, 38)  ELISA (4, 6, 10, 12, 14, 20, 21, 25, 28, 29, 30, 34, 41, 44, 47, 48, 51, 52, 53, 64, 71, 83, 87, 90, 98, 105, 110)  Multiplex-Assay (91, 117)  qRT-PCR (36)  Radioimmunoassay (26) |
| **sTNF-RI** | Tumor necrosis factor receptor superfamily member 1A, CD120a | **↑** (10)  ▬ (20, 46, 48, 110, 111) | ▬ (20, 48) | P.d.R. (111) |  | ELISA (10, 20, 46, 48, 111) |
| **sTNF-RII** | Tumor necrosis factor receptor superfamily member 1B, CD120b | ▬ (39, 48, 111) | ▬ (48) | ▬ (39, 111) | **↑** (54) | ELISA (39, 48)  Multiplex Assay (54) |
| **TGF-β** | Transforming growth factor beta, homodimer consisting of either two subunits TGF-beta 1, TGF-beta 2 or TGF-beta 3. | **↑** (32, 94, 106)  **↓** (3, 35, 79)  ▬ (40, 109)  P.d.R.(13) | **↑** (40, 94, 102, 103)  **↓** (47)  ▬ (64) |  | **↓** (47) | Cell-based bioassay (32, 94)  ELISA (13, 35, 40, 47, 64, 102, 103, 109)  WB (3, 79) |
| **Protein** | **Synonyms** | **Alzheimer´s disease (AD)** | | **Mild cognitive impairment (MCI)** | | **Method** |
|  |  | **Plasma/Serum** | **CSF** | **Plasma/Serum** | **CSF** |  |
| **TRAIL** | Tumor necrosis factor ligand superfamily member 10, Apo-2 ligand (Apo-2L), TNF-related apoptosis-inducing ligand, CD253 | ▬ (17, 88) |  | ▬ (17) |  | ELISA (17, 88) |
| **TRAIL-R4** | Tumor necrosis factor receptor superfamily member 10D, TNF-related apoptosis-inducing ligand receptor 4, TRAIL receptor 4, CD264 | ▬ (1, 6) |  |  |  | Cytokine Array (1, 6) |
| **VCAM-1** | Vascular cell adhesion protein 1, CD106 | **↑** (63, 87, 118)  ▬ (17) |  | ▬ (17) |  | ELISA (17, 87)  Multiplex Assay (63, 118) |
| **VEGF** | Vascular endothelial growth factor A | **↑** (15) | **↑** (102)  ▬ (64) |  | **↓** (54) | Cytokine Array (15)  ELISA (64, 102)  Multiplex Assay (54) |

Supplementary References List:

**1**. Ray S, Britschgi M, Herbert C, et al. Classification and prediction of clinical Alzheimer's diagnosis based on plasma signaling proteins. Nat Med 2007;13:1359-1362.

**2**. Westin K, Buchhave P, Nielsen H, Minthon L, Janciauskiene S, Hansson O. CCL2 is associated with a faster rate of cognitive decline during early stages of Alzheimer's disease. PLoS One 2012;7:e30525.

**3**. Mocali A, Cedrola S, Della Malva N, et al. Increased plasma levels of soluble CD40, together with the decrease of TGF beta 1, as possible differential markers of Alzheimer disease. Exp Gerontol 2004;39:1555-1561.

**4**. Rao JS, Kellom M, Kim HW, Rapoport SI, Reese EA. Neuroinflammation and synaptic loss. Neurochem Res 2012;37:903-910.

**5**. Ait-ghezala G, Abdullah L, Volmar CH, et al. Diagnostic utility of APOE, soluble CD40, CD40L, and Abeta1-40 levels in plasma in Alzheimer's disease. Cytokine 2008;44:283-287.

**6**. Bjorkqvist M, Ohlsson M, Minthon L, Hansson O. Evaluation of a previously suggested plasma biomarker panel to identify Alzheimer's disease. PLoS One 2012;7:e29868.

**7**. Ojala J, Alafuzoff I, Herukka SK, van Groen T, Tanila H, Pirttila T. Expression of interleukin-18 is increased in the brains of Alzheimer's disease patients. Neurobiol Aging 2009;30:198-209.

**8**. Licastro F, Pedrini S, Caputo L, et al. Increased plasma levels of interleukin-1, interleukin-6 and alpha-1-antichymotrypsin in patients with Alzheimer's disease: peripheral inflammation or signals from the brain? J Neuroimmunol 2000;103:97-102.

**9**. Magaki S, Mueller C, Dickson C, Kirsch W. Increased production of inflammatory cytokines in mild cognitive impairment. Exp Gerontol 2007;42:233-240.

**10**. De Luigi A, Fragiacomo C, Lucca U, Quadri P, Tettamanti M, Grazia De Simoni M. Inflammatory markers in Alzheimer's disease and multi-infarct dementia. Mech Ageing Dev 2001;122:1985-1995.

**11**. Kim SM, Song J, Kim S, et al. Identification of peripheral inflammatory markers between normal control and Alzheimer's disease. BMC Neurol 2011;11:51.

**12**. Bermejo P, Martin-Aragon S, Benedi J, et al. Differences of peripheral inflammatory markers between mild cognitive impairment and Alzheimer's disease. Immunol Lett 2008;117:198-202.

**13**. Motta M, Imbesi R, Di Rosa M, Stivala F, Malaguarnera L. Altered plasma cytokine levels in Alzheimer's disease: correlation with the disease progression. Immunol Lett 2007;114:46-51.

**14**. Zuliani G, Guerra G, Ranzini M, et al. High interleukin-6 plasma levels are associated with functional impairment in older patients with vascular dementia. Int J Geriatr Psychiatry 2007;22:305-311.

**15**. Corsi MM, Licastro F, Porcellini E, et al. Reduced plasma levels of P-selectin and L-selectin in a pilot study from Alzheimer disease: relationship with neuro-degeneration. Biogerontology 2011;12:451-454.

**16**. Galimberti D, Schoonenboom N, Scarpini E, Scheltens P. Chemokines in serum and cerebrospinal fluid of Alzheimer's disease patients. Ann Neurol 2003;53:547-548.

**17**. Lee KS, Chung JH, Lee KH, Shin MJ, Oh BH, Hong CH. Bioplex analysis of plasma cytokines in Alzheimer's disease and mild cognitive impairment. Immunol Lett 2008;121:105-109.

**18**. Galimberti D, Schoonenboom N, Scheltens P, et al. Intrathecal chemokine synthesis in mild cognitive impairment and Alzheimer disease. Arch Neurol 2006;63:538-543.

**19**. Singh VK, Guthikonda P. Circulating cytokines in Alzheimer's disease. J Psychiatr Res 1997;31:657-660.

**20**. Richartz E, Stransky E, Batra A, et al. Decline of immune responsiveness: a pathogenetic factor in Alzheimer's disease? J Psychiatr Res 2005;39:535-543.

**21**. Kassner SS, Bonaterra GA, Kaiser E, et al. Novel systemic markers for patients with Alzheimer disease? - a pilot study. Curr Alzheimer Res 2008;5:358-366.

**22**. Laske C, Stellos K, Eschweiler GW, Leyhe T, Gawaz M. Decreased CXCL12 (SDF-1) plasma levels in early Alzheimer's disease: a contribution to a deficient hematopoietic brain support? J Alzheimers Dis 2008;15:83-95.

**23**. O'Bryant SE, Hobson V, Hall JR, et al. Brain-derived neurotrophic factor levels in Alzheimer's disease. J Alzheimers Dis 2009;17:337-341.

**24**. Reale M, Kamal MA, Velluto L, Gambi D, Di Nicola M, Greig NH. Relationship between inflammatory mediators, Abeta levels and ApoE genotype in Alzheimer disease. Curr Alzheimer Res 2012;9:447-457.

**25**. Baranowska-Bik A, Bik W, Wolinska-Witort E, et al. Plasma beta amyloid and cytokine profile in women with Alzheimer's disease. Neuro Endocrinol Lett 2008;29:75-79.

**26**. Alvarez XA, Franco A, Fernandez-Novoa L, Cacabelos R. Blood levels of histamine, IL-1 beta, and TNF-alpha in patients with mild to moderate Alzheimer disease. Mol Chem Neuropathol 1996;29:237-252.

**27**. Blum-Degen D, Muller T, Kuhn W, Gerlach M, Przuntek H, Riederer P. Interleukin-1 beta and interleukin-6 are elevated in the cerebrospinal fluid of Alzheimer's and de novo Parkinson's disease patients. Neurosci Lett 1995;202:17-20.

**28**. Bruunsgaard H, Andersen-Ranberg K, Jeune B, Pedersen AN, Skinhoj P, Pedersen BK. A high plasma concentration of TNF-alpha is associated with dementia in centenarians. J Gerontol A Biol Sci Med Sci 1999;54:M357-364.

**29**. Tarkowski E, Blennow K, Wallin A, Tarkowski A. Intracerebral production of tumor necrosis factor-alpha, a local neuroprotective agent, in Alzheimer disease and vascular dementia. J Clin Immunol 1999;19:223-230.

**30**. Maes M, DeVos N, Wauters A, et al. Inflammatory markers in younger vs elderly normal volunteers and in patients with Alzheimer's disease. J Psychiatr Res 1999;33:397-405.

**31**. Angelis P, Scharf S, Mander A, Vajda F, Christophidis N. Serum interleukin-6 and interleukin-6 soluble receptor in Alzheimer's disease. Neurosci Lett 1998;244:106-108.

**32**. Chao CC, Ala TA, Hu S, et al. Serum cytokine levels in patients with Alzheimer's disease. Clin Diagn Lab Immunol 1994;1:433-436.

**33**. van Duijn CM, Hofman A, Nagelkerken L. Serum levels of interleukin-6 are not elevated in patients with Alzheimer's disease. Neurosci Lett 1990;108:350-354.

**34**. Chen R, Yin Y, Zhao Z, et al. Elevation of serum TNF-alpha levels in mild and moderate Alzheimer patients with daytime sleepiness. J Neuroimmunol 2012;244:97-102.

**35**. Juraskova B, Andrys C, Holmerova I, et al. Transforming growth factor beta and soluble endoglin in the healthy senior and in Alzheimer's disease patients. J Nutr Health Aging 2010;14:758-761.

**36**. Kester MI, van der Flier WM, Visser A, Blankenstein MA, Scheltens P, Oudejans CB. Decreased mRNA expression of CCL5 [RANTES] in Alzheimer's disease blood samples. Clin Chem Lab Med 2012;50:61-65.

**37**. Forlenza OV, Diniz BS, Talib LL, et al. Increased serum IL-1beta level in Alzheimer's disease and mild cognitive impairment. Dement Geriatr Cogn Disord 2009;28:507-512.

**38**. Marksteiner J, Kemmler G, Weiss EM, et al. Five out of 16 plasma signaling proteins are enhanced in plasma of patients with mild cognitive impairment and Alzheimer's disease. Neurobiol Aging 2011;32:539-540.

**39**. Hernanz A, De la Fuente M, Navarro M, Frank A. Plasma aminothiol compounds, but not serum tumor necrosis factor receptor II and soluble receptor for advanced glycation end products, are related to the cognitive impairment in Alzheimer's disease and mild cognitive impairment patients. Neuroimmunomodulation 2007;14:163-167.

**40**. Rota E, Bellone G, Rocca P, Bergamasco B, Emanuelli G, Ferrero P. Increased intrathecal TGF-beta1, but not IL-12, IFN-gamma and IL-10 levels in Alzheimer's disease patients. Neurol Sci 2006;27:33-39.

**41**. Alvarez A, Cacabelos R, Sanpedro C, Garcia-Fantini M, Aleixandre M. Serum TNF-alpha levels are increased and correlate negatively with free IGF-I in Alzheimer disease. Neurobiol Aging 2007;28:533-536.

**42**. Galimberti D, Fenoglio C, Lovati C, et al. Serum MCP-1 levels are increased in mild cognitive impairment and mild Alzheimer's disease. Neurobiol Aging 2006;27:1763-1768.

**43**. Lindberg C, Chromek M, Ahrengart L, Brauner A, Schultzberg M, Garlind A. Soluble interleukin-1 receptor type II, IL-18 and caspase-1 in mild cognitive impairment and severe Alzheimer's disease. Neurochem Int 2005;46:551-557.

**44**. Solerte SB, Cravello L, Ferrari E, Fioravanti M. Overproduction of IFN-gamma and TNF-alpha from natural killer (NK) cells is associated with abnormal NK reactivity and cognitive derangement in Alzheimer's disease. Ann N Y Acad Sci 2000;917:331-340.

**45**. O'Bryant SE, Waring SC, Hobson V, et al. Decreased C-reactive protein levels in Alzheimer disease. J Geriatr Psychiatry Neurol 2010;23:49-53.

**46**. Hasegawa Y, Sawada M, Ozaki N, Inagaki T, Suzumura A. Increased soluble tumor necrosis factor receptor levels in the serum of elderly people. Gerontology 2000;46:185-188.

**47**. Tarkowski E, Andreasen N, Tarkowski A, Blennow K. Intrathecal inflammation precedes development of Alzheimer's disease. J Neurol Neurosurg Psychiatry 2003;74:1200-1205.

**48**. Lanzrein AS, Johnston CM, Perry VH, Jobst KA, King EM, Smith AD. Longitudinal study of inflammatory factors in serum, cerebrospinal fluid, and brain tissue in Alzheimer disease: interleukin-1beta, interleukin-6, interleukin-1 receptor antagonist, tumor necrosis factor-alpha, the soluble tumor necrosis factor receptors I and II, and alpha1-antichymotrypsin. Alzheimer Dis Assoc Disord 1998;12:215-227.

**49**. Lepara O, Alajbegovic A, Zaciragic A, et al. Elevated serum homocysteine level is not associated with serum C-reactive protein in patients with probable Alzheimer's disease. J Neural Transm 2009;116:1651-1656.

**50**. Mashayekhi F, Hadavi M, Vaziri HR, Naji M. Increased acidic fibroblast growth factor concentrations in the serum and cerebrospinal fluid of patients with Alzheimer's disease. J Clin Neurosci 2010;17:357-359.

**51**. Engelborghs S, De Brabander M, De Cree J, et al. Unchanged levels of interleukins, neopterin, interferon-gamma and tumor necrosis factor-alpha in cerebrospinal fluid of patients with dementia of the Alzheimer type. Neurochem Int 1999;34:523-530.

**52**. Fillit H, Ding WH, Buee L, et al. Elevated circulating tumor necrosis factor levels in Alzheimer's disease. Neurosci Lett 1991;129:318-320.

**53**. Garlind A, Brauner A, Hojeberg B, Basun H, Schultzberg M. Soluble interleukin-1 receptor type II levels are elevated in cerebrospinal fluid in Alzheimer's disease patients. Brain Res 1999;826:112-116.

**54**. Craig-Schapiro R, Kuhn M, Xiong C, et al. Multiplexed immunoassay panel identifies novel CSF biomarkers for Alzheimer's disease diagnosis and prognosis. PLoS One 2011;6:e18850.

**55**. Hampel H, Schoen D, Schwarz MJ, et al. Interleukin-6 is not altered in cerebrospinal fluid of first-degree relatives and patients with Alzheimer's disease. Neurosci Lett 1997;228:143-146.

**56**. Hampel H, Teipel SJ, Padberg F, et al. Discriminant power of combined cerebrospinal fluid tau protein and of the soluble interleukin-6 receptor complex in the diagnosis of Alzheimer's disease. Brain Res 1999;823:104-112.

**57**. Harigaya Y, Shoji M, Nakamura T, Matsubara E, Hosoda K, Hirai S. Alpha 1-antichymotrypsin level in cerebrospinal fluid is closely associated with late onset Alzheimer's disease. Intern Med 1995;34:481-484.

**58**. Licastro F, Morini MC, Polazzi E, Davis LJ. Increased serum alpha 1-antichymotrypsin in patients with probable Alzheimer's disease: an acute phase reactant without the peripheral acute phase response. J Neuroimmunol 1995;57:71-75.

**59**. Licastro F, Masliah E, Pedrini S, Thal LJ. Blood levels of alpha-1-antichymotrypsin and risk factors for Alzheimer's disease: effects of gender and apolipoprotein E genotype. Dement Geriatr Cogn Disord 2000;11:25-28.

**60**. Martinez M, Frank A, Hernanz A. Relationship of interleukin-1 beta and beta 2-microglobulin with neuropeptides in cerebrospinal fluid of patients with dementia of the Alzheimer type. J Neuroimmunol 1993;48:235-240.

**61**. Martinez M, Fernandez-Vivancos E, Frank A, De la Fuente M, Hernanz A. Increased cerebrospinal fluid fas (Apo-1) levels in Alzheimer's disease. Relationship with IL-6 concentrations. Brain Res 2000;869:216-219.

**62**. Marz P, Heese K, Hock C, et al. Interleukin-6 (IL-6) and soluble forms of IL-6 receptors are not altered in cerebrospinal fluid of Alzheimer's disease patients. Neurosci Lett 1997;239:29-32.

**63**. Doecke JD, Laws SM, Faux NG, et al. Blood-Based Protein Biomarkers for Diagnosis of Alzheimer Disease. Arch Neurol 2012:1-8.

**64**. Blasko I, Lederer W, Oberbauer H, et al. Measurement of thirteen biological markers in CSF of patients with Alzheimer's disease and other dementias. Dement Geriatr Cogn Disord 2006;21:9-15.

**65**. Pirttila T, Mehta PD, Frey H, Wisniewski HM. Alpha 1-antichymotrypsin and IL-1 beta are not increased in CSF or serum in Alzheimer's disease. Neurobiol Aging 1994;15:313-317.

**66**. Yamada K, Kono K, Umegaki H, et al. Decreased interleukin-6 level in the cerebrospinal fluid of patients with Alzheimer-type dementia. Neurosci Lett 1995;186:219-221.

**67**. Licastro F, Parnetti L, Morini MC, et al. Acute phase reactant alpha 1-antichymotrypsin is increased in cerebrospinal fluid and serum of patients with probable Alzheimer disease. Alzheimer Dis Assoc Disord 1995;9:112-118.

**68**. Hampel H, Sunderland T, Kotter HU, et al. Decreased soluble interleukin-6 receptor in cerebrospinal fluid of patients with Alzheimer's disease. Brain Res 1998;780:356-359.

**69**. Angelucci F, Spalletta G, di Iulio F, et al. Alzheimer's disease (AD) and Mild Cognitive Impairment (MCI) patients are characterized by increased BDNF serum levels. Curr Alzheimer Res 2010;7:15-20.

**70**. Li G, Peskind ER, Millard SP, et al. Cerebrospinal fluid concentration of brain-derived neurotrophic factor and cognitive function in non-demented subjects. PLoS One 2009;4:e5424.

**71**. Angelopoulos P, Agouridaki H, Vaiopoulos H, et al. Cytokines in Alzheimer's disease and vascular dementia. Int J Neurosci 2008;118:1659-1672.

**72**. Buchhave P, Janciauskiene S, Zetterberg H, Blennow K, Minthon L, Hansson O. Elevated plasma levels of soluble CD40 in incipient Alzheimer's disease. Neurosci Lett 2009;450:56-59.

**73**. Murase K, Nabeshima T, Robitaille Y, Quirion R, Ogawa M, Hayashi K. NGF level of is not decreased in the serum, brain-spinal fluid, hippocampus, or parietal cortex of individuals with Alzheimer's disease. Biochem Biophys Res Commun 1993;193:198-203.

**74**. Lieberman J, Schleissner L, Tachiki KH, Kling AS. Serum alpha 1-antichymotrypsin level as a marker for Alzheimer-type dementia. Neurobiol Aging 1995;16:747-753.

**75**. DeKosky ST, Ikonomovic MD, Wang X, et al. Plasma and cerebrospinal fluid alpha1-antichymotrypsin levels in Alzheimer's disease: correlation with cognitive impairment. Ann Neurol 2003;53:81-90.

**76**. Han Y, Jia J, Jia XF, Qin W, Wang S. Combination of plasma biomarkers and clinical data for the detection of sporadic Alzheimer's disease. Neurosci Lett 2012;516:232-236.

**77**. Licastro F, Pedrini S, Davis LJ, et al. Alpha-1-antichymotrypsin and oxidative stress in the peripheral blood from patients with probable Alzheimer disease: a short-term longitudinal study. Alzheimer Dis Assoc Disord 2001;15:51-55.

**78**. O'Bryant SE, Hobson VL, Hall JR, et al. Serum brain-derived neurotrophic factor levels are specifically associated with memory performance among Alzheimer's disease cases. Dement Geriatr Cogn Disord 2011;31:31-36.

**79**. De Servi B, La Porta CA, Bontempelli M, Comolli R. Decrease of TGF-beta1 plasma levels and increase of nitric oxide synthase activity in leukocytes as potential biomarkers of Alzheimer's disease. Exp Gerontol 2002;37:813-821.

**80**. Dicou E, Vermersch P, Penisson-Besnier I, Dubas F, Nerriere V. Anti-NGF autoantibodies and NGF in sera of Alzheimer patients and in normal subjects in relation to age. Autoimmunity 1997;26:189-194.

**81**. Laske C, Stransky E, Leyhe T, et al. Stage-dependent BDNF serum concentrations in Alzheimer's disease. J Neural Transm 2006;113:1217-1224.

**82**. Laske C, Stransky E, Leyhe T, et al. BDNF serum and CSF concentrations in Alzheimer's disease, normal pressure hydrocephalus and healthy controls. J Psychiatr Res 2007;41:387-394.

**83**. Bonotis K, Krikki E, Holeva V, Aggouridaki C, Costa V, Baloyannis S. Systemic immune aberrations in Alzheimer's disease patients. J Neuroimmunol 2008;193:183-187.

**84**. Teunissen CE, Lutjohann D, von Bergmann K, et al. Combination of serum markers related to several mechanisms in Alzheimer's disease. Neurobiol Aging 2003;24:893-902.

**85**. Laske C, Stellos K, Hoffmann N, et al. Higher BDNF serum levels predict slower cognitive decline in Alzheimer's disease patients. Int J Neuropsychopharmacol 2011;14:399-404.

**86**. Inspector M, Aharon-Perez J, Glass-Marmor L, Miller A. Matrix metalloproteinase-9, its tissue inhibitor(TIMP)-1 and CRP in Alzheimer's disease. Eur Neurol 2005;53:155-157.

**87**. Zuliani G, Cavalieri M, Galvani M, et al. Markers of endothelial dysfunction in older subjects with late onset Alzheimer's disease or vascular dementia. J Neurol Sci 2008;272:164-170.

**88**. Genc S, Egrilmez MY, Yaka E, et al. TNF-related apoptosis-inducing ligand level in Alzheimer's disease. Neurol Sci 2009;30:263-267.

**89**. Laske C, Stransky E, Hoffmann N, et al. Macrophage colony-stimulating factor (M-CSF) in plasma and CSF of patients with mild cognitive impairment and Alzheimer's disease. Curr Alzheimer Res 2010;7:409-414.

**90**. Yasutake C, Kuroda K, Yanagawa T, Okamura T, Yoneda H. Serum BDNF, TNF-alpha and IL-1beta levels in dementia patients: comparison between Alzheimer's disease and vascular dementia. Eur Arch Psychiatry Clin Neurosci 2006;256:402-406.

**91**. Choi C, Jeong JH, Jang JS, et al. Multiplex analysis of cytokines in the serum and cerebrospinal fluid of patients with Alzheimer's disease by color-coded bead technology. J Clin Neurol 2008;4:84-88.

**92**. Lawlor BA, Swanwick GR, Feighery C, Walsh JB, Coakley D. Acute phase reactants in Alzheimer's disease. Biol Psychiatry 1996;39:1051-1052.

**93**. Mulder SD, Heijst JA, Mulder C, et al. CSF levels of PSA and PSA-ACT complexes in Alzheimer's disease. Ann Clin Biochem 2009;46:477-483.

**94**. Chao CC, Hu S, Frey WH, 2nd, Ala TA, Tourtellotte WW, Peterson PK. Transforming growth factor beta in Alzheimer's disease. Clin Diagn Lab Immunol 1994;1:109-110.

**95**. Laske C, Stellos K, Stransky E, Leyhe T, Gawaz M. Decreased plasma levels of granulocyte-colony stimulating factor (G-CSF) in patients with early Alzheimer's disease. J Alzheimers Dis 2009;17:115-123.

**96**. Helmy AA, Naseer MM, Shafie SE, Nada MA. Role of interleukin 6 and alpha-globulins in differentiating Alzheimer and vascular dementias. Neurodegener Dis 2012;9:81-86.

**97**. Cojocaru IM, Cojocaru M, Miu G, Sapira V. Study of interleukin-6 production in Alzheimer's disease. Rom J Intern Med 2011;49:55-58.

**98**. Jia JP, Meng R, Sun YX, Sun WJ, Ji XM, Jia LF. Cerebrospinal fluid tau, Abeta1-42 and inflammatory cytokines in patients with Alzheimer's disease and vascular dementia. Neurosci Lett 2005;383:12-16.

**99**. Rosler N, Wichart I, Jellinger KA. Clinical significance of neurobiochemical profiles in the lumbar cerebrospinal fluid of Alzheimer's disease patients. J Neural Transm 2001;108:231-246.

**100**. Gomez-Tortosa E, Gonzalo I, Fanjul S, et al. Cerebrospinal fluid markers in dementia with lewy bodies compared with Alzheimer disease. Arch Neurol 2003;60:1218-1222.

**101**. Galimberti D, Venturelli E, Fenoglio C, et al. Intrathecal levels of IL-6, IL-11 and LIF in Alzheimer's disease and frontotemporal lobar degeneration. J Neurol 2008;255:539-544.

**102**. Tarkowski E, Issa R, Sjogren M, et al. Increased intrathecal levels of the angiogenic factors VEGF and TGF-beta in Alzheimer's disease and vascular dementia. Neurobiol Aging 2002;23:237-243.

**103**. Zetterberg H, Andreasen N, Blennow K. Increased cerebrospinal fluid levels of transforming growth factor-beta1 in Alzheimer's disease. Neurosci Lett 2004;367:194-196.

**104**. Correa JD, Starling D, Teixeira AL, Caramelli P, Silva TA. Chemokines in CSF of Alzheimer's disease patients. Arq Neuropsiquiatr 2011;69:455-459.

**105**. Ozturk C, Ozge A, Yalin OO, et al. The diagnostic role of serum inflammatory and soluble proteins on dementia subtypes: correlation with cognitive and functional decline. Behav Neurol 2007;18:207-215.

**106**. Malaguarnera L, Motta M, Di Rosa M, Anzaldi M, Malaguarnera M. Interleukin-18 and transforming growth factor-beta 1 plasma levels in Alzheimer's disease and vascular dementia. Neuropathology 2006;26:307-312.

**107**. Matsubara E, Amari M, Shoji M, et al. Serum concentration of alpha 1-antichymotrypsin is elevated in patients with senile dementia of the Alzheimer type. Prog Clin Biol Res 1989;317:707-714.

**108**. Cacabelos R, Alvarez XA, Franco-Maside A, Fernandez-Novoa L, Caamano J. Serum tumor necrosis factor (TNF) in Alzheimer's disease and multi-infarct dementia. Methods Find Exp Clin Pharmacol 1994;16:29-35.

**109**. Rodriguez-Rodriguez E, Sanchez-Juan P, Mateo I, et al. Serum levels and genetic variation of TGF-beta1 are not associated with Alzheimer's disease. Acta Neurol Scand 2007;116:409-412.

**110**. De Luigi A, Pizzimenti S, Quadri P, et al. Peripheral inflammatory response in Alzheimer's disease and multiinfarct dementia. Neurobiol Dis 2002;11:308-314.

**111**. Diniz BS, Teixeira AL, Ojopi EB, et al. Higher serum sTNFR1 level predicts conversion from mild cognitive impairment to Alzheimer's disease. J Alzheimers Dis 2010;22:1305-1311.

**112**. Bonaccorso S, Lin A, Song C, et al. Serotonin-immune interactions in elderly volunteers and in patients with Alzheimer's disease (DAT): lower plasma tryptophan availability to the brain in the elderly and increased serum interleukin-6 in DAT. Aging (Milano) 1998;10:316-323.

**113**. Kalman J, Juhasz A, Laird G, et al. Serum interleukin-6 levels correlate with the severity of dementia in Down syndrome and in Alzheimer's disease. Acta Neurol Scand 1997;96:236-240.

**114**. Vom Berg J, Prokop S, Miller KR, et al. Inhibition of IL-12/IL-23 signaling reduces Alzheimer's disease-like pathology and cognitive decline. Nat Med 2012;18:1812-1819.

**115**. Zhang R, Miller RG, Madison C, et al. Systemic immune system alterations in early stages of Alzheimer's disease. J Neuroimmunol 2013;256:38-42.

**116**. Huang L, Jia J, Liu R. Decreased serum levels of the angiogenic factors VEGF and TGF-beta1 in Alzheimer's disease and amnestic mild cognitive impairment. Neurosci Lett 2013;550:60-63.

**117**. Soares HD, Chen Y, Sabbagh M, Roher A, Schrijvers E, Breteler M. Identifying early markers of Alzheimer's disease using quantitative multiplex proteomic immunoassay panels. Ann N Y Acad Sci 2009;1180:56-67.

**118**. Doecke JD, Laws SM, Faux NG, et al. Blood-based protein biomarkers for diagnosis of Alzheimer disease. Arch Neurol 2012;69:1318-1325.
